# Evolution of Highly Pathogenic H5N1 Avian Influenza Viruses in Vietnam between 2001 and 2007

**DOI:** 10.1371/journal.pone.0003462

**Published:** 2008-10-21

**Authors:** Xiu-Feng Wan, Tung Nguyen, C. Todd Davis, Catherine B. Smith, Zi-Ming Zhao, Margaret Carrel, Kenjiro Inui, Hoa T. Do, Duong T. Mai, Samadhan Jadhao, Amanda Balish, Bo Shu, Feng Luo, Michael Emch, Yumiko Matsuoka, Stephen E. Lindstrom, Nancy J. Cox, Cam V. Nguyen, Alexander Klimov, Ruben O. Donis

**Affiliations:** 1 Influenza Division, Centers for Disease Control and Prevention, Atlanta, Georgia, United States of America; 2 School of Biology, Georgia Institute of Technology, Atlanta, Georgia, United States of America; 3 Department of Animal Health, National Centre for Veterinary Diagnostics, Hanoi, Vietnam; 4 Department of Geography, University of North Carolina, Chapel Hill, North Carolina, United States of America; 5 Food and Agriculture Organization of Vietnam, Hanoi, Vietnam; 6 School of Computing, Clemson University, Clemson, South Carolina, United States of America; University of Maryland, United States of America

## Abstract

Highly pathogenic avian influenza (HPAI) H5N1 viruses have caused dramatic economic losses to the poultry industry of Vietnam and continue to pose a serious threat to public health. As of June 2008, Vietnam had reported nearly one third of worldwide laboratory confirmed human H5N1 infections. To better understand the emergence, spread and evolution of H5N1 in Vietnam we studied over 300 H5N1 avian influenza viruses isolated from Vietnam since their first detection in 2001. Our phylogenetic analyses indicated that six genetically distinct H5N1 viruses were introduced into Vietnam during the past seven years. The H5N1 lineage that evolved following the introduction in 2003 of the A/duck/Hong Kong/821/2002-like viruses, with clade 1 hemagglutinin (HA), continued to predominate in southern Vietnam as of May 2007. A virus with a clade 2.3.4 HA newly introduced into northern Vietnam in 2007, reassorted with pre-existing clade 1 viruses, resulting in the emergence of novel genotypes with neuraminidase (NA) and/or internal gene segments from clade 1 viruses. A total of nine distinct genotypes have been present in Vietnam since 2001, including five that were circulating in 2007. At least four of these genotypes appear to have originated in Vietnam and represent novel H5N1 viruses not reported elsewhere. Geographic and temporal analyses of H5N1 infection dynamics in poultry suggest that the majority of viruses containing new genes were first detected in northern Vietnam and subsequently spread to southern Vietnam after reassorting with pre-existing local viruses in northern Vietnam. Although the routes of entry and spread of H5N1 in Vietnam remain speculative, enhanced poultry import controls and virologic surveillance efforts may help curb the entry and spread of new HPAI viral genes.

## Introduction

The emergence of highly pathogenic avian influenza (HPAI) H5N1 virus as a human pathogen occurred in 1997 in Hong Kong Special Administrative Region (SAR) [1]. Since that outbreak, H5N1 has spread among birds out of East Asia across Eurasia and as far west as England and West Africa, threatening further spread into the American and Australian continents. H5N1 is primarily a pathogen of poultry; more than 200 million poultry died or have been culled because of this virus [2]. The virus is also of great concern for public health because it has caused several hundred human infections, suggesting that it could become transmissible among humans and cause a pandemic. Approximately 60% of the 385 human infections (as of 19 June, 2008) have been fatal [3], but very few have been transmitted from person to person [4]. Infections are usually acquired by contact with H5N1 infected poultry or poultry products, and the H5N1 viruses isolated from human cases are often virtually identical to isolates from poultry [5].

A global effort is underway to control or eradicate H5N1 in poultry and prevent human exposure, both of which may also reduce the risk of pandemic emergence. The scientific rationale for these programs is provided by ecologic, virologic, epidemiologic, and immunologic studies. In particular, molecular and functional characterization of H5N1 viruses from poultry will help inform development and implementation of public health control measures involving diagnosis, immunization and antiviral drug therapy.

The putative ancestor of the currently circulating H5N1 viruses is A/goose/Guangdong/1/96 (Gs/GD/1/96), named after the province in southern China which is also thought to be the epicenter of H5N1 [6], [7]. However, contemporary H5N1 viruses carry only the hemagglutinin (HA) gene derived from the Gs/GD/1/96 lineage [8]. The other seven genes of contemporary H5N1 viruses were acquired from other avian influenza viruses by genetic reassortment [9]. The Gs/GD/1/96 HA gene has evolved extensively in the past decade, diverging into multiple clades (termed clades 0–9) [10]. Viruses with clade 1 HA genes were predominant in poultry in Southeast Asia (Vietnam, Laos, Cambodia, Thailand, China) until 2005.

In 2005, unprecedented outbreaks of H5N1 in wild migratory birds at Qinghai Lake in western China apparently facilitated its spread to more than 30 countries in Europe, the Middle East and Africa [11]. These outbreaks were caused by viruses with a distinct HA that belongs to clade 2.2 [12]. Following the westward spread of HA Clade 2.2 viruses, other viruses with a divergent HA gene, termed clade 2.3.4 emerged in southern China and began to spread in this region [13].

Although HA gene evolution merits much attention because of its central role in antigenic drift and interaction with the host, the remaining seven viral genes are also critical to inform disease control efforts as they contribute to antiviral drug susceptibility, host range and virulence, among other phenotypic properties [9]. The phylogenetic characterization of the eight viral genes defines what is termed the viral genotype. At least twenty-one distinct H5N1 genotypes have been reported during the past eleven years in southern China [9], [14], [15], [16].

Vietnam (VN) is one of the countries most affected by H5N1 outbreaks in poultry and has accumulated 106 laboratory-confirmed human cases since 2003. Recurring epizootics of H5N1 were reported in poultry from 2003 until 2005 [2]. Little H5N1 virus activity was reported in late 2006, but the virus re-emerged in 2007 when poultry outbreaks of H5N1 were reported in more than 20 provinces in Vietnam, and at least thirteen laboratory confirmed human cases were detected [2], [3]. Although the H5N1 viruses that circulated in Vietnam from 2003–2005 had clade 1 HA genes, many viruses isolated in 2007 had newly introduced clade 2.3.4 HA genes [17]. In order to investigate the origin of H5N1 viruses isolated in Vietnam and to identify potential reassortant genotypes within the country, we characterized H5N1 isolates from different regions collected between 2001 and 2007. This report demonstrates that at least six clades of H5N1 have emerged in Vietnam since 2001 and provides evidence of reassortment between clade 1 and clade 2 viruses. Further analyses suggest that viruses were initially introduced into northern Vietnam and then spread into southern Vietnam. This systematic analysis of the evolution of HPAI H5N1 in Vietnam will help provide a comprehensive approach towards more efficient disease control and prevention for both poultry and humans.

## Results

### H5N1 Avian Influenza Virus Surveillance in Vietnam

The nationwide H5N1 surveillance program launched in 2004 by the Government of Vietnam and implemented by the National Centre for Veterinary Diagnostics (NCVD), in collaboration with the Vietnam Department of Animal Health (DAH) and DAH Regional Animal Health offices, involved the collection and analysis of 20,567 samples from 38 provinces between that year and May of 2007. A total of 2,111 positive specimens were detected by RT-PCR or real-time RT-PCR specific for H5 HA RNA (Table 1). Although this represents an overall average rate of 10.3% H5-positive samples, the rate of positive samples ranged from 3.1% for 2006 to 22.7% for 2004. All specimens received by NCVD that were identified as H5-positive by RT-PCR or real-time RT-PCR were selected for virus isolation in embryonated chicken eggs. A total of 158 H5N1 viruses were isolated and their genomes sequenced. The phylogenetic analyses performed included 174 additional H5N1 viruses from Vietnam whose genomic sequences were available in public databases, as well as four H5N1 viruses collected during 2007 poultry outbreaks in Laos that were isolated at NCVD (Table S1).

**Table 1:** Surveillance of HPAI H5N1 in birds in Vietnam from 2004 to 2007.

	2004^a^	2005^a^	2006^b^	2007^b^	Total
Total samples	2,272	13,889	64	4,342	20,567
Positive Samples	515 (22.7%)^c^	1,317 (9.4%)	2 (3.1%)	277 (6.4%)	2,111 (10.3%)
Number of provinces^d^	28	34	12	35	38

a Results from RT-PCR.

b Results from real-time RT-PCR.

c Percentage of samples positive out of total collected.

d See Table S3 for a specific description of the geographic location of HPAI H5N1 viruses from Vietnam.

### Introduction of H5N1 HA genes into Vietnam

**Figure 1:**
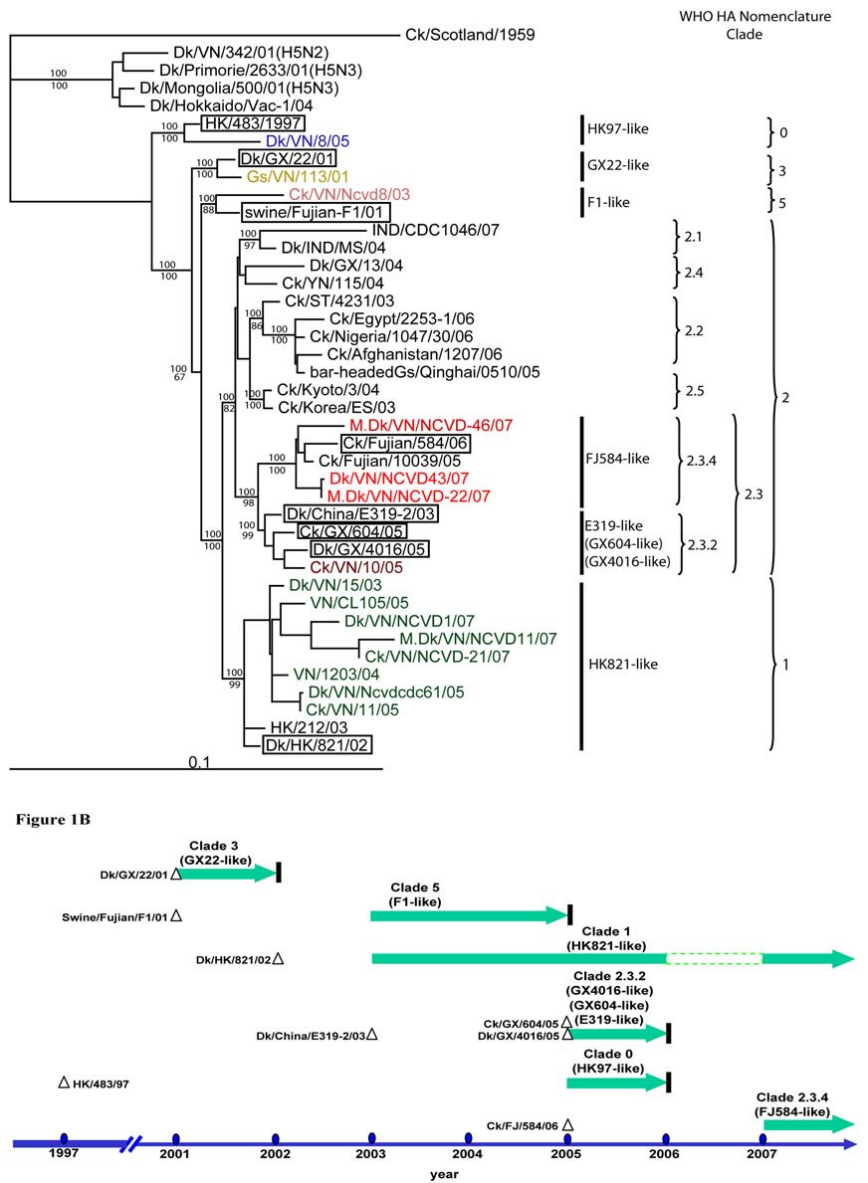
Multiple introductions of HPAI H5N1 into Vietnam from 2001 to 2007. (A) Phylogenetic tree of subtype H5 HA genes from avian influenza viruses. The phylogenetic tree was constructed by Maximum Likelihood using GARLI version 0.951 [34] by selecting GTR+I+G model from Modeltest 3.7 [37]. Posterior probabilities and bootstrap values were given above and below branches, respectively. The phylogenetic tree was rooted by Ck/Scotland/59. Each avian influenza gene precursor was defined with the shortest phylogenetic distance to the Vietnam lineages: HK97-, HK821-, E319-, FJ584-, GX22-, and F1-, GX604-, and GX4016-like (Table 2). The viruses isolated from Vietnam are marked in colors other than black. The predicted precursor viruses are shown in boxes. A detailed tree showing the HK821-like lineage can be viewed in Figure S2. (B) Yearly H5N1 isolation in Vietnam by HA clade. The horizontal arrows denote the year in which the viruses were detected. The vertical line to the right of an arrow denotes that the particular HA clade was not detected in subsequent years. Triangles depict the time of isolation of the postulated precursor virus. These six distinct HA clades were apparently introduced into Vietnam between 2001 and 2007 approximately.

In order to investigate the origin of the HA genes of H5N1 viruses isolated in Vietnam prior to May 2007, we first constructed their phylogeny. Six different HA clades were identified and designated according to the recently described nomenclature system for the highly pathogenic H5N1 viruses as well as by potential precursors: clade 0, HK97-like (HK/483/97); clade 1, HK821-like (Dk/HK/821/02); clade 2.3.2, E319-like (Dk/China/E319-2/03); clade 2.3.4, FJ584-like (Ck/Fujian/584/05); clade 3, GX22-like (Dk/GX/22/01); clade 5, F1-like (swine/Fujian/F1/01) (Figure 1A; Table 2) [10]. The six HA clades that we identified clustered with distinct precursor viruses isolated previously in mainland China and Hong Kong SAR. These precursor viruses were likely to be ancestral to each of the Vietnam virus lineages. Thus, we postulate that there have been at least six independent introductions of highly pathogenic H5N1 virus into Vietnam (Figure 1A and 1B).

**Table 2:** Putative precursor viruses of HPAI H5N1 from Vietnam.

Precursor virus	Abbreviation
HK/483/97	HK97-like
Dk/HK/821/02	HK821-like
Dk/China/E319-2/03	E319-like
Dk/GX/4016/05	GX4016-like
Ck/GX/604/05	GX406-like
Ck/Fujian/584/06	FJ584-like
Dk/GX/22/01	GX22-like
Sw/Fujian/F1/01	F1-like

**Figure 2:**
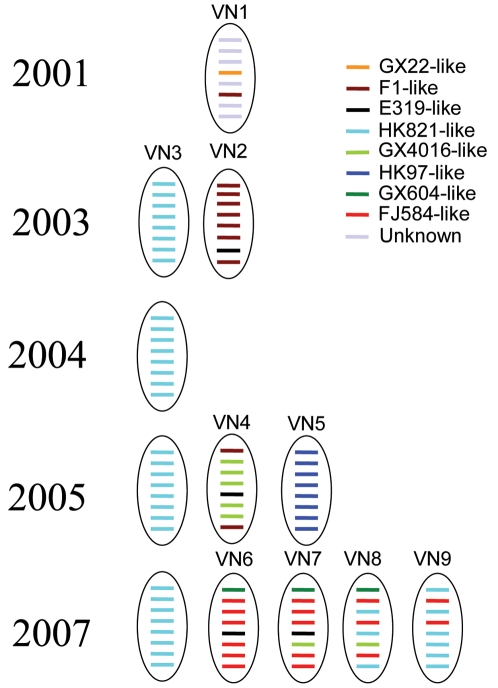
Emerging HPAI H5N1 genotypes from introduction and reassortment in Vietnam from 2001 to 2007. Each segment was marked in a different color corresponding to its putative precursor virus. The segment numbers are ordered 1 to 8 from top to bottom. Table 3 shows the H5N1 viruses from Vietnam that represent each genotype and other information.

The timing of H5N1 clade isolation from poultry indicated that some lineages were present for one year or less, while others continued to circulate in Vietnam for more than 3 years. For instance, following their first detection in 2003, clade 5 viruses circulated in Vietnam for approximately 1 year and were replaced by clade 1 viruses in 2004. The clade 1 viruses first detected in late 2003 continued to circulate until 2007. In addition, other viral clades co-circulated in the region in 2005; e.g., clade 0 and clade 2.3.2 (Figure 1B, Figure 2).

In 2006, there were very few outbreaks reported in Vietnam possibly due to increased use of poultry vaccines after August, 2005. Our surveillance detected two positive samples among 64 tested using real-time RT-PCR. However, no H5N1 viruses were isolated from these positive samples. A sharp decrease in poultry outbreaks throughout Vietnam during 2006 led to a transition into a passive mode of surveillance for H5N1 and accounted for the low number of samples collected for testing in comparison to other years (Table 1). However, the multiple H5N1 outbreaks in poultry and isolation of clade 1 viruses in 2007 strongly suggests that these viruses were circulating at low levels during 2006 or that closely related viruses were re-introduced from neighboring countries.

### Phylogenetic Analyses of NA and Internal Genes Revealed an Abundant Genome Segment Pool in Vietnam

**Figure 3:**
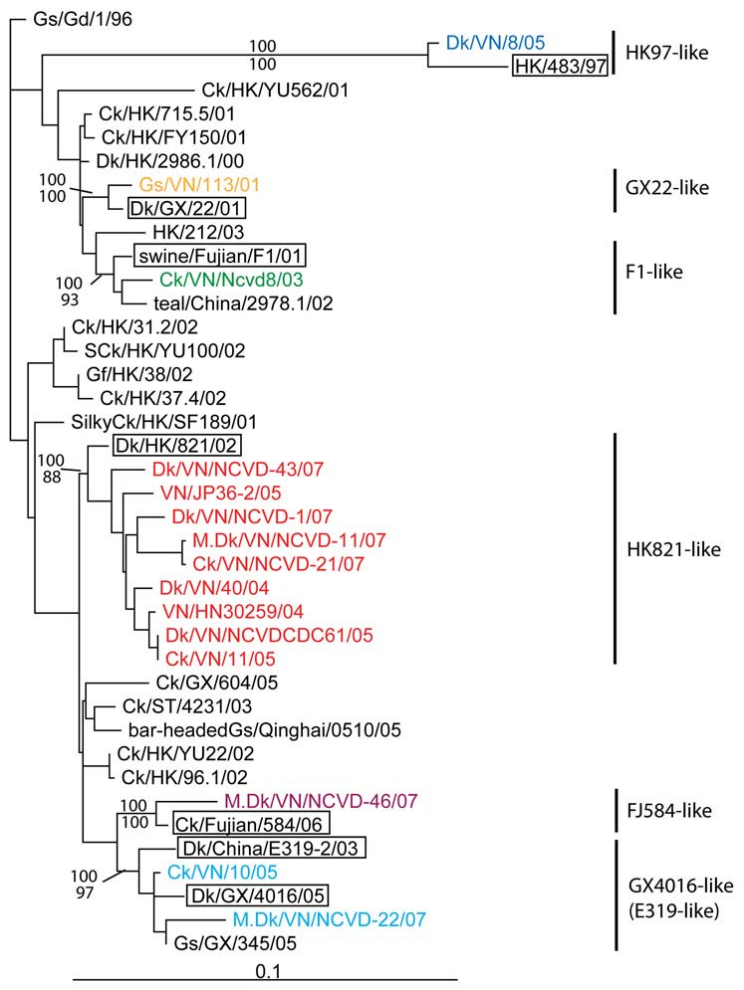
Phylogenetic tree of the NA gene of HPAI H5N1 viruses. The phylogenetic tree was constructed by Maximum Likelihood using GARLI version 0.951 [34] by selecting GTR+G model from Modeltest 3.7 [37]. Posterior probabilities and bootstrap values were given above and below branches, respectively. The tree was rooted by Gs/GD/1/96. The predicted precursor viruses were shown in boxes.

Phylogenetic analyses of the NA and the so-called “internal gene” segments (PB2, PB1, PA, NP, M, NS) of all available H5N1 viruses from Vietnam (>300 viruses) were performed to establish the diversity of the influenza gene pool present in Vietnam. Phylogenetic trees for each gene segment revealed the potential precursor virus for each lineage of the NA and internal gene segments (Figure 3 and Figure S1A-H). Each lineage was defined as a group in a tree having a bootstrap and/or Bayesian posterior probability value over 60. The trees revealed that the NA and internal genes of Vietnam isolates were likely derived from at least one of the eight putative precursor viruses defined in Table 2. The phylogenies of PB2, NP, M, and NS genes denoted the presence of four distinct lineages, while the PB1 and PA genes were derived from five different lineages, and the NA gene from six lineages. The diversity of lineages for each gene segment of Vietnam H5N1 isolates and the likely precursor viruses for each lineage are shown in Table 3 and Figure S1A-H. Collectively, these results indicate that the NA as well as the remaining six internal genes originated from at least eight potential ancestor viruses, each of which contributed one or more gene segments to the overall H5N1 gene pool in Vietnam (Table 2 and Table 3). The high degree of sequence similarity between genes of each Vietnam isolate and putative precursor viral genes, as shown in Table 3, suggests that Vietnam isolates likely descended from the putative precursor virus or a closely related virus already present in Vietnam. The co-evolution of segments within each genome was analyzed by comparing the relative position of each isolate in the phylogenetic tree (tree topology) for each gene segment. Analysis of all publicly available HPAI H5N1 gene segments in comparison to Vietnam H5N1 gene segments revealed reassortment events between Vietnam viruses and H5N1 viruses detected earlier in neighboring China and Hong Kong SAR. The individual gene segment trees also revealed that gene segments of Vietnam isolates from a given clade did not always co-evolve with other members of that clade. For instance, Dk/VN/NCVD-43/07 had an HA gene derived from clade 2.3.4 viruses but an NA gene from HK821-like viruses, whose HAs belong to clade 1 viruses (Figure 1A and Figure 3). Similar examples of reassortment between viruses from distinct clades were also identified in clade 5 and clade 2.3.2 viruses.

**Table 3:** Genotypes identified in Vietnam from 2001 to 2007.

Virus	Virus segment								Year	Genotype
	HA^a^	NA	PB2	PB1	PA	NP	MP	NS		
Gs/VN/113/01	clade 3 GX22-like; 98.8^b^	GX22-like; 99.1	ND^c^	ND	ND	ND	ND	ND	2001	VN1
Ck/VN/NCVD8/03	clade 5 F1-like; 97.7	F1-like; 98.7	F1-like; 99.0 (GX4016-like; 98.7)^d^	F1-like; 97.8	F1-like; 97.8	F1-like; 98.5	E319-like; 99.2 (GX4016-like; 99.3)	F1-like; 98.7 (GX4016-like; 97.3)	2003	VN2
Dk/VN/NCVD-7/07	clade 1 HK821-like; 97.5	HK821-like; 97.6	HK821-like; 98.4	HK821-like; 98.3	HK821-like; 97.8	HK821-like; 98.4	HK821-like; 98.3	HK821-like; 97.3	2003–2007	VN3
Ck/VN/10/05	clade 2.3.2 GX4016-like; 98.9 (E319-like; 98.6) (GX604-like; 98.6)	GX4016-like; 99.1 (E319-like; 98.7)	F1-like; 98.6 (GX4016-like; 99.1)	GX4016-like; 99.2	GX4016-like; 99.3	E319-like; 99.1 (FJ584-like; 98.0) (GX4016-like; 98.6)	E319-like; 98.9 (GX4016-like; 99.6)	F1-like;97.6 (GX4016-like;98.9)	2005	VN4
Dk/VN/8/05	clade 0 HK97-like; 97.8	HK97-like; 97.7	HK97-like; 97.6	HK97-like; 99.4	HK97-like; 98.2	HK97-like; 98.2	HK97-like; 99.1	HK97-like; 99.7	2005	VN5
Ck/VN/NCVD-44/07	clade 2.3.4 FJ584-like; 97.9	FJ584-like; 98.0	GX604-like; 98.3 (E319-like; 97.2)	FJ584-like; 98.7 (E319-like; 98.1)	FJ584-like; 98.4	E319-like; 99.1 (FJ584-like; 97.7) (GX4016-like; 98.7)	FJ584-like; 99.0	FJ584-like; 98.7	2007	VN6
Ck/VN/NCVD-20/07	clade 2.3.4 FJ584-like; 98.1	GX4016-like; 97.6 (E319-like; 97.3)	GX604-like; 98.3 (E319-like; 97.3)	FJ584-like; 98.8 (E319-like; 98.2)	FJ584-like; 98.0	E319-like; 99.1 (FJ584-like; 97.7) (GX4016-like; 98.6)	FJ584-like; 98.8	FJ584-like; 98.3	2007	VN7
M.Dk/VN/NCVD-22/07	clade 2.3.4 FJ584-like; 98.2	GX4016-like; 97.5 (E319-like; 97.3)	GX604-like; 97.9 (E319-like; 97.1)	FJ584-like; 98.7 (E319-like; 98.1)	HK821-like; 96.8	HK821-like; 98.6	FJ584-like; 98.6	HK821-like; 98.2	2007	VN8
Dk/VN/NCVD-43/07	clade 2.3.4 FJ584-like; 97.3	HK821-like; 98.1	HK821-like; 97.2	FJ584-like; 96.5 (E319-like; 96.3)	HK821-like; 98.3	HK821-like; 98.6	HK821-like; 98.9	HK821-like; 97.3	2007	VN9

a Based on the WHO HA nomenclature for HPAI H5N1 [10].

b The number indicates the nucleotide sequence identity percentile between each gene segment of a representative Vietnam H5N1 virus and the corresponding gene from the putative progenitor virus.

c ND denotes the associated segment sequence is not available.

d The precursor virus outside the parenthesis is the primary precursor virus, which had a closer phylogenetic relationship to a specific Vietnam virus lineage than the other potential precursor virus shown within the parenthesis. If a lineage had more than one potential precursor, only one virus was marked as the primary precursor-like virus and other closely related viruses were listed within parentheses.

### Emerging Genotypes from Reassortment

The diverse gene segment lineages identified in H5N1 viruses isolated in Vietnam between 2001 and 2007 provide a large gene pool for reassortment. By combining the phylogenetic information from all the genes of each isolate, we were able to determine the number of viral genotypes present in Vietnam from 2001 to 2007 (Figure S1). As shown in Figure 2, at least 9 genotypes were introduced or have emerged in Vietnam since 2001 (termed VN1-VN9), five of which were detected in 2007. Genotypes VN6 through VN9 had the clade 2.3.4 HA gene (FJ584-like) first detected in Vietnam in 2007, and at least one segment from a different virus present in Vietnam before 2007 (i.e., derived from HK821-, E319-, GX604-, or GX4016-like viruses). Genotypes VN8 and VN9, in particular, provide evidence that clade 1 and clade 2 viruses have reassorted (Figure 2). Because clade 2.3.4 viruses were first identified in China in 2005 [13], we postulate that reassortment yielding genotypes VN6 through VN9 occurred after the clade 2.3.4 viruses were introduced into Vietnam. It is important to point out that only VN3 genotype viruses were isolated from human H5N1 infections in Vietnam between 2004 and 2005 [3]. Although there were no human infections detected in 2006, eight new cases were confirmed in 2007, five of which were fatal. It will be critical to investigate whether the new human cases have been caused by viruses belonging to the VN3 genotype or one of the other newly emerging genotypes.

### Spatial and Temporal Distribution of H5N1 Genotypes

**Figure 4:**
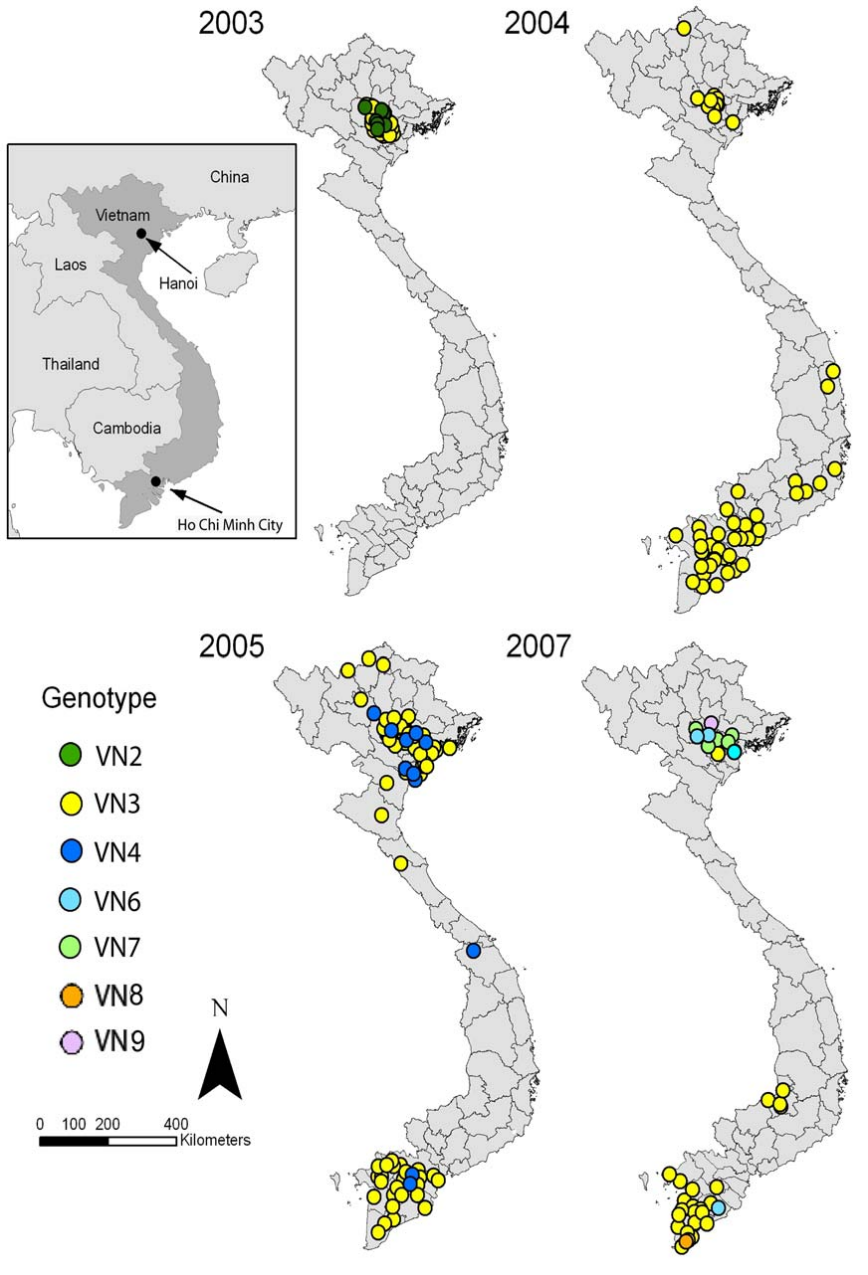
Geographic distribution of the genotypes identified for 142 H5N1 viruses isolated in Vietnam from 2003 to 2007. The viral genotype of each H5N1 isolate was mapped chronologically to show the time of genotype isolation in certain regions of Vietnam. VN5 was not shown in this figure since the geographic locations of these viruses were not known. The strain name, genotype, and collection location of each isolate are listed in Table S1.

In order to trace the time and location of H5N1 genotype emergence and spread within Vietnam, we plotted the geographical location of each genotype onto a map of the country. While the specific collection sites of the VN1 genotype virus within Vietnam was not known, VN2 genotype virus, which contains all F1-like (clade 5) segments except M, was first detected in 2003. During 2003, both the VN2 and VN3 genotypes were detected only in northern Vietnam despite equal sampling of birds in southern Vietnam during the same time period. In 2004, however, VN3 genotype viruses were isolated in both southern and northern Vietnam [18]. Although this genotype was still predominant in 2005, two unrelated genotypes (VN4 and VN5) were detected (Figure 4 and Table S2). Genotype VN5, which contained genes related to the HK97-like clade 0 viruses, seemed to quickly disappear. In contrast, the VN4 genotype viruses (clade 2.3.2 HA) spread throughout both northern and southern Vietnam (Figure 4 and Figure S1A). Genotype VN4 viruses were probably generated by reassortment between E319-, GX604-, and GX4016-like viruses (clade 2.3.2) introduced into Vietnam in 2005 and an earlier pre-existing genotype. Although the VN2 genotype was only detected in 2003, reassortment between VN2 genotype viruses and the newly introduced clade 2.3.2 viruses may have given rise to genotype VN4 (PB2 and NS segments are shared between these viruses) (Figure 2 and Figures S1C and S1H).

Despite markedly lower levels of H5N1 detection in 2006, perhaps as a result of a successful vaccination campaign coupled with a decline in sampling due to a transition to passive surveillance in poultry, five new genotypes were detected in 2007 (VN6-VN9). Each of these genotypes contained the HA gene from the clade 2.3.4 viruses, which were introduced into Vietnam in 2007. In addition, they contained gene segments from other viruses isolated earlier in Vietnam, suggesting that these genotypes emerged locally from reassortment of the newly introduced clade 2.3.4 viruses with pre-existing ones. Reassortment yielding genotypes VN6, VN7, and VN9 appear to have occurred near the Hanoi area as the majority of viruses from these genotypes have been detected in this region of Vietnam (Figure 4). Of these new genotypes, VN6 viruses were also detected in southern Vietnam (Soc Trang Province), which may indicate a migration of this genotype from northern to southern Vietnam. The VN8 genotype was only detected in southern Vietnam and is likely a reassortant between VN7 and VN3, since the latter was predominant in southern Vietnam during the early months of 2007. Thus, the emergence of new genotypes appears to correlate with the co-circulation of viral genotypes in specific regions of Vietnam.

### Genetic and antigenic evolution of clade 1 hemagglutinins

The VN3 genotype was predominant throughout Vietnam and neighboring countries between 2003 and 2005. The large size of Vietnam's bird population and the high rates of infection in this period were conducive to short viral generation times and rapid evolution. This genotype continued to circulate in 2007, but it was isolated with lower frequency than the VN6 genotype in northern Vietnam (Figure 4, Table S2). The phylogeny of clade 1 HA genes indicated their progressive divergence into three sub-lineages; HK821P, HK821α, and HK821β (Figure S2). The HK821P sub-lineage included precursor viruses that evolved into two additional sub-lineages containing HK821α and HK821β. HK821P-like viruses have been present in both northern and southern Vietnam since 2003. The HK821α sub-lineage was predominant in northern Vietnam in 2005, while the HK821β sub-lineage was predominant in southern Vietnam during 2007. The divergence of these two sub-lineages within the country along geographical lines suggests the presence of other factors, such as poultry trade routes, geographical boundaries and/or migratory routes, which may restrict the co-circulation of these groups of viruses in Vietnam.

To complement the genetic analysis of HK821α and HK821β, we compared their amino acid sequences at positions with known functional roles. Interestingly, the receptor binding site of both HK821α and HK821β contained residue 123P (corresponding to residue 127 in H3 influenza A virus numbering) instead of 123S, which was found in the HK821P and FJ584-like virus sub-lineages [19]. Additional positive selection analyses of HK821-like HA genes demonstrated that codon 123 was positively selected [20].

### Two Major Antigenic Groups Circulated in Vietnam During 2007

**Figure 5:**
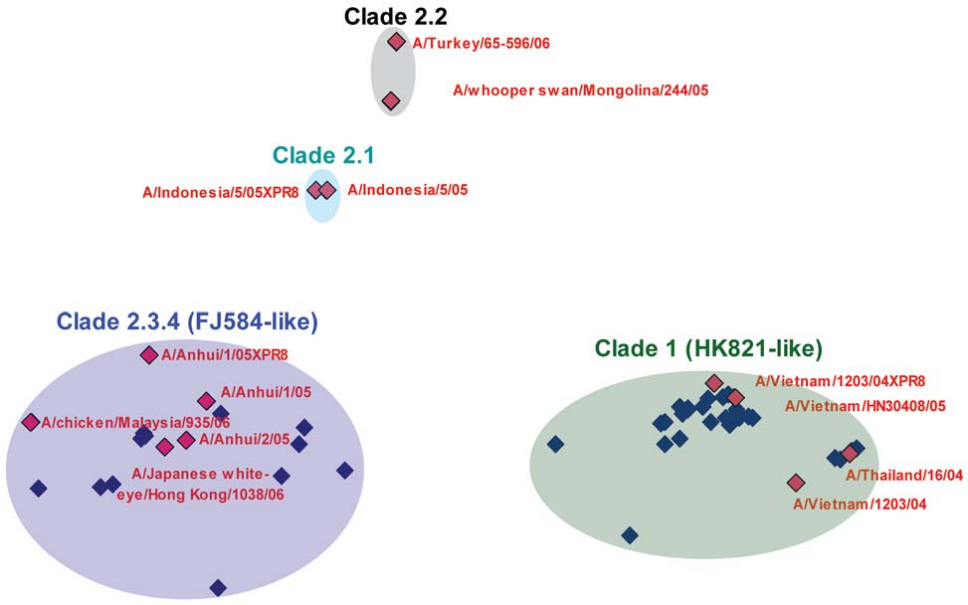
Two major antigenic groups circulated in Vietnam during 2007. There are 13 WHO reference antigens (marked in red diamonds) and each of the 40 HPAI H5N1 Vietnam viruses isolated in 2007 (marked in blue diamonds; Table S1). Multidimensional scaling analysis was used to characterize the antigenicity of these viruses based on HI data using SPSS 15.0 (SPSS, Inc.) with the default s-stress convergence value of 0.001.

A panel of ferret antisera to the WHO reference subtype H5N1 viruses (clades 1, 2.1.3, 2.2 and 2.3.4) was used in hemagglutination inhibition assays with the 40 HPAI H5N1 viruses isolated during 2007. The homologous clade HI titers were at least four-fold higher than those in the heterologous reactions, indicating a strong correlation of genetic and antigenic characteristics. A/chicken/Fujian/584/2006-like (clade 2.3.4) and A/duck/Hong Kong/821/2002-like (clade 1) viruses had distinct properties, as illustrated in Figure 5. The FJ584-like viruses were antigenically related to clade 2.3.4 viruses, such as A/Anhui/1/05, A/chicken/Maylasia/935/06, and A/Japanese white-eye/Hong Kong/1038/06. The HK821-like viruses were antigenically related to clade 1 viruses, such as A/Vietnam/1203/04. In order to identify potential amino acid substitutions, which may influence the antigenicity of specific clades, we determined the diversity of HA1 consensus sequence residues between each clade. The HA1 sequence differences between clade 1, 2.1, 2.2 and 2.3 are listed in Table 4. Although a specific correlation between amino acid differences and antigenicity was not determined in this study, the diversity of residues between each clade clearly influences antigenicity and the results demonstrate the existence of antigenically distinct H5N1 viruses co-circulating in parts of Vietnam.

**Table 4:** Residue diversities between the HA1 sequences of Vietnam H5 hemagglutinins from H5N1 avian influenza viruses.

Virus	Residue position^a^										
	86	94	155	174	181	212	227	269	282	310	322
clade 1	V/A/I	D/N/V	S/N	V	P	R/K	E	L	M	R	Q
clade 2.1	A/T	S/N	S/N	V	P	K	E	L	M	R	Q
clade 2.2	A	N/D	N/D	V	P	K	E	L	I	R	Q
clade 2.3	A/V	N	N/D	I/V	S/P	K	D	V	I/M	K	L/Q

a Residue position denotes the amino acid position in the mature HA protein sequence.

## Discussion

HPAI H5N1 viruses were first identified in Vietnam in 2001 and have since caused repeated poultry outbreaks throughout the country leading to dramatic economic losses to the nation's poultry industry. Early introductions of H5N1 in Vietnam (in 2001) led to only sporadic detection of the virus in domestic birds [21]. However, clade 1 viruses introduced in 2003 caused multiple poultry outbreaks in Vietnam in 2004 and 2005, which continued to occur in 2007 and 2008 [18]. Additionally, Vietnam has recorded at least 106 human cases of H5N1 infection resulting in 52 deaths since 2003 [3]. In order to better understand the ecology and molecular epidemiology of H5N1 viruses present in Vietnam, a national surveillance program was initiated by NCVD to detect and characterize HPAI H5N1 viruses.

During the 2003 H5N1 avian influenza outbreaks in Vietnam, the predominant viruses identified belonged to the so-called Z genotype, which were first identified in China in 2002 (referred to as the VN3 genotype in this study) [22]. Although the route of introduction of this genotype into Vietnam remains unclear, its close phylogenetic relationship to isolates from China suggests a relatively direct transmission link between Vietnam and China [23]. The VN3 genotype viruses have been circulating in both northern and southern Vietnam since their introduction and have replaced previously circulating genotypes detected in 2001 and early 2003. Interestingly, the earlier genotypes identified in Vietnam, referred to as VN1 and VN2, possessed HA genes belonging to clade 3 and 5 and have not been identified since 2001 and 2003, respectively. However, our results show that the F1-like PB2 and NS segments from the VN2 genotype were present in Vietnam isolates in 2005 (genotype VN4) suggesting that viruses containing the F1-like segments from the VN2 genotype continued to circulate in Vietnam after 2003 and may have reassorted with the clade 2.3.2 viruses that were introduced into Vietnam before or during 2005 to generate the VN4 genotype.

The VN3 genotype, which was predominate in northern and southern Vietnam throughout 2004 and 2005, has continued to circulate at least through the early part of 2007 despite introduction of other genotypes in 2005 and 2007 (Figure 2). The NA gene segment of VN4 viruses (clade 2.3.2) was detected in circulating 2007 genotypes (VN7-VN8), while the NP gene segment from VN4 was seen in VN6 and VN7 only. This finding suggests that viruses containing gene segments from VN4 continued to circulate or were re-introduced into Vietnam after 2005.

Four distinct genotypes containing clade 2.3.4 (FJ584-like) HA genes were identified in Vietnam after May 2007: VN6-VN9. Interestingly, these viruses were primarily collected in northern Vietnam and appear to be replacing the VN3 genotype in this part of the country (Figure 4). Additional surveillance will reveal if the clade 2.3.4 HA genotypes will become predominant in the south as observed in the north.

The diversity of genotypes present in Vietnam also provides a pluripotent reservoir from which an epidemic or pandemic strain may arise. The discovery of four previously unidentified genotypes (VN4, VN7, VN8 and VN9) confirms previous observations that reassortment between H5N1 viruses occurs frequently. Reassortment between clade 1 viruses (HK821-like) and clade 2.3.4 viruses (FJ584-like) and between clade 2.3.2 (GX4016-like) and clade 5 (F1-like) viruses suggests a high level of genetic compatibility between viruses with diverse parental genotypes. In addition, the emergence of three new genotypes (VN7, VN8, and VN9) following the introduction of clade 2.3.4 viruses (VN6) in 2007, and the concurrent eclipse of VN3 in northern Vietnam suggests that these genotypes may have a selective advantage. One obvious possibility is the antigenic novelty of the clade 2.3.4 HA. Another example of the possible advantage of antigenic diversity is the expansion of HK821β-like viruses that emerged in southern Vietnam in 2007 (Figure S2). It remains to be seen what impact these mutations may have on the ability of these viruses to infect humans in Vietnam or elsewhere, especially given that the majority of the human H5N1 isolates from 2003 to 2007 have been caused by clade 1 viruses (Figure 1 and Figure S2).

As noted in previous studies, some viruses may have spread bi-directionally between countries [17]. For example, cross-border poultry trade between Vietnam and China may have led to the introduction of clade 2.3.4 and other lineages into Vietnam. While the precursor viruses identified in this study were first detected in mainland China and Hong Kong SAR, it remains to be seen if viral genotypes that appear to have emerged in Vietnam will spread into other countries. Results from this study also identified reassortants between distinct viral genotypes, which indicate their co-circulation and suggest common mechanisms of spread. In addition, it is possible that some of the internal gene segments identified were derived from non-H5N1 avian subtypes (i.e., H9, H7, etc.) known to circulate in Vietnam.

New genotypes were first identified in northern provinces of Vietnam and appear to have gradually spread to southern provinces via unknown mechanisms. The majority of isolates were collected from specific locations in Vietnam with high human population densities, especially in areas around Hanoi and Ho Chi Minh City, which also contain most of the country's industrial and backyard poultry flocks. Although sampling outside of these large cities was less intensive, it is possible that avian influenza transmission from northern to southern Vietnam occurs via direct poultry trade routes between major population centers in each region [24]. Alternatively, the apparent northern to southern spread of virus may correspond to trade routes along the Mekong River from Laos to Vietnam. Cross-border transmission between Cambodia and southern Vietnam may also influence the pattern of genotype emergence identified by this study. Strengthening H5N1 surveillance programs in Vietnam and neighboring countries will fill gaps in knowledge of avian influenza ecology and mechanisms of spread.

Inactivated H5N1 and H5N2 vaccines have been used in Vietnam since August of 2005 to control H5N1 viruses [2]. In 2006, only a few suspected H5N1 outbreaks were reported in Vietnam and no viruses were isolated, which might be a consequence of vaccination programs [25], [26]. However, after December of 2006, two waves of avian influenza outbreaks were reported across 31 provinces in Vietnam [2]. The H5N1 viruses isolated in this study after 2006 were phylogenetically close to those isolated in 2004 and 2005 (>99% nucleotide identity for most genes) suggesting that 2007 viruses were descendants of earlier Vietnam viruses. Thus, it is likely that H5N1 viruses were not completely eradicated from Vietnam and bordering countries in 2006, but escaped detection due perhaps to a transition towards passive surveillance in poultry.

In summary, our studies of viruses isolated in Vietnam during 2001–2007 demonstrate that multiple viral introductions and reassortment events have occurred frequently in Vietnam leading to the emergence of at least four novel genotypes. Thus, disease prevention and control must be applied inside the country and at the borders, to prevent introduction of new HPAI H5N1 strains, which expand the H5N1 viral gene pool. Strengthening vaccination and associated surveillance programs in poultry would help decrease the current avian influenza virus genetic reservoir in Vietnam, and H5N1 viruses in particular, thus reducing the threat to public health.

## Materials and Methods

### Poultry surveillance and sample collection

The National Centre for Veterinary Diagnostics (NCVD, Hanoi, Vietnam) surveillance program was initiated in 2004 in collaboration with the Vietnam Department of Animal Health (DAH) and DAH Regional Animal Health offices to detect H5N1 infections in poultry. Collection sites included poultry farms, backyard flocks, and live bird markets in 38 provinces representing regions in both northern and southern Vietnam. Cloacal swab specimens were collected in tubes with virus transport medium. Swab samples, as well as sick or dead bird specimens, were submitted to NCVD and regional laboratories. Tissue samples collected from slaughtered sick birds included the bronchus, lung, kidney, spleen, and brain. Tissue specimens were placed immediately on ice in sterile plastic bags and later stored at −80°C. From 2001–2003, samples were screened for H5N1 virus by inoculation in 10–11 day old embryonated chicken eggs (ECEs) for virus amplification. Allantoic fluid from ECEs was tested for the presence of virus by performing a hemagglutination assay using chicken red blood cells. In 2004 and 2005, an influenza subtype H5 specific RT-PCR test was used to identify samples containing H5N1 viral sequences. Since 2006, real-time RT-PCR has been used to identify samples containing H5N1 RNA, which were then selected for inoculation into ECEs for isolation.

### Virus isolation, RNA extraction, and genomic sequencing

Following transfer of swab samples and/or viral isolates to the Centers for Disease Control and Prevention, Atlanta, GA, highly pathogenic H5N1 viruses were isolated from positive samples by infection of either 10–11 day old ECEs or MDCK cells. A total of 158 H5N1 viruses were isolated from 2001 to May of 2007. RNA isolation and sequencing procedures were described previously [27]. Briefly, viral RNA was extracted from virus-infected allantoic fluid or cell culture supernatants with the Qiagen RNeasy extraction kit, and a one-step RT-PCR kit (Qiagen) was used to amplify genomic segments as full-length or overlapping fragments. The amplicons were sequenced on an automated Applied Biosystems 3730 system using cycle sequencing dye terminator chemistry (Perkin-Elmer, Foster City, CA). Each gene segment sequence was submitted to GenBank for deposition (See Table S1 for accession numbers). Primer sequences are available upon request. All infectious viruses were handled in biosafety level 3 containment, including enhancements required by the U.S. Department of Agriculture and the Select Agents program (http://www.cdc.gov/od/ohs/biosfty/bmbl5/bmbl5toc.htm).

### Datasets used for phylogenetic analysis

In addition to the 158 isolates collected by NCVD and sequenced for this study (Table S1), for the analyses we also included an additional 174 publicly accessible isolates collected in Vietnam from 2001–2007. Of the 332 isolates, 192 were analyzed for genotyping purposes because they had complete or nearly complete gene sequences for all eight gene segments (Table S1). The provincial location in Vietnam where each virus-containing sample was collected is shown in Table S1 and is plotted on a map of Vietnam to show approximate location of specific genotypes identified by this study (Figure 4). ArcGIS 9.1 (ESRI, Inc.) was used to map the geographic location of each genotype. The analyses also included four H5N1 viruses collected by NCVD during 2007 poultry outbreaks in Laos. Isolates were collected over many regions of both northern and southern Vietnam with nearly equal distribution in the north and south (Table S3). In addition to the gene sequences of H5N1 Vietnam isolates, datasets used for phylogenetic analyses also contained all of the 44,398 influenza virus sequences available from the Influenza Virus Resource database including all H1 and H3 genes, as well as other influenza subtype genes [28], which were retrieved from the database in August of 2007.

### Phylogenetic analysis

To identify the potential precursor viruses for each Vietnam isolate, we applied our newly developed Gene In Network (GIN) method [29]. Briefly, GIN first measured the evolutionary distances between isolates using the Complete Composition Vector (CCV) approach [29], [30], [31]. Then GIN identified influenza virus modules, clusters of viruses with small evolutionary distances, using a local optimization program based on the thresholds derived from Bayesian analysis [29]. Unlike the conventional phylogenetic tree construction approach, GIN is able to efficiently analyze a larger number of sequences. Inclusion of all available 44,398 influenza viral sequences during this analysis facilitated systematic identification of the progenitor genes of Vietnam H5N1 viruses. Following identification of related groups of viruses using GIN, selected isolate sequences from the clusters of larger groups were subsequently analyzed by conventional phylogenetic methods as described below. Potential precursor viruses to Vietnam isolates identified in this study were selected based on three main criteria: (1) the precursor viral gene must have the shortest phylogenetic distance to the homologous genes of its associated Vietnam isolate(s); (2) the precursor virus contributes the majority of the donor gene segments to the Vietnam isolate(s) in comparison to other closely related viruses; (3) the specific sub-lineage with a precursor virus and its associated Vietnam isolates must have a statistical confidence value (bootstrap value or posterior probability, described in the following section) over 60.

Phylogenetic inferences relied on Maximum Parsimony (MP) and Neighbor-Joining (NJ) methods using PAUP* 4.0 Beta [32], as described earlier [33]. Maximum Likelihood (ML) tree estimation was evaluated using GARLI version 0.951 [34]. Bayesian trees were estimated by MrBayes version 3.1.2 with 1 million generations, sampling every 100 generations, using the default heating parameter, in two runs. The consensus trees were calculated using the allcompat option from the final 10,001 trees from each run. Tree topologies were confirmed between each of these three methods. Bootstrapping support for tree topologies were performed using NJ methods implemented in PAUP* 4.0 Beta with 1,000 replicates. When Bayesian trees were estimated, the posterior probability for each split was generated using the MrBayes sumt option with a 25% burnin [35], [36]. These posterior probabilities were used as an alternative measure of clade assignment support. The nucleotide substitution models for ML and NJ methods were selected using MODELTEST 3.7 [37]. The positive selection analyses for HK821-like isolates and other 2007 VN isolates were conducted using PAML [38]. Control and log files for all stand-alone programs used in these analyses and other methodological materials are available upon request.

### Antigenic analysis

Antigenic characterization of HPAI H5N1 isolated in Vietnam during 2007 was performed using the hemagglutinin inhibition (HI) assay with ferret antisera to the WHO reference H5 subtype viruses, including A/Turkey/65-596/06, A/whooper swan/Mongolina/244/05, A/Indonesia/5/05×PR8, A/Indonesia/5/05, A/Anhui/1/05×PR8, A/chicken/Malaysia/935/06, A/Anhui/1/05, A/Japanese white-eye/Hong Kong/1038/06, A/Anhui/2/05, A/Vietnam/1203/04×PR8, A/Vietnam/1203/04, A/Vietnam/HN30408/05, and A/Thailand/16/04. The HI assay was performed with a starting serum dilution of 1∶5. The multidimensional scaling analysis was performed using SPSS 15.0 (SPSS, Inc.) to analyze HI titers in order to correlate data between 2007 Vietnam H5N1 viruses and WHO H5N1 reference antigens. The HI data for each antigen-antiserum pair was normalized by dividing the maximum HI value for that antiserum. The pairwise *Euclidean* distances between antigens were calculated using these normalized HI data. Within the multidimensional scaling analysis, the s-stress convergence value was set to the default value 0.001, the minimum s-stress value was set to the default value 0.05, and the maximum iteration was set to 100.

### Accession numbers

Gene sequences generated during this study have been deposited into the GenBank database, and accession numbers can be found in Table S1.

## Supporting Information

Table S1 Summary of the HPAI H5N1 viruses analyzed in this study.
(0.86 MB PDF)

Table S2 Number of H5N1 isolates collected and percentage of each identified genotype in Vietnam per year.
(0.01 MB PDF)

Table S3 Geographic origin of HPAI H5N1 viruses from Vietnam.
(0.01 MB PDF)

Figure S1 Phylogenetic analysis of the H5N1 highly pathogenic avian influenza viruses isolated in Vietnam. Figures A-H represent segments HA, NA, PB2, PB1, PA, NP, MP, and NS, respectively. Posterior probabilities and bootstrap values are given above and below branches. A red dot was marked beside each predicted precursor virus. Previously reported genotypes, such as A, B, C, D, E, G, X0, X1, X2, X3, Y, V, Z, and Z+ [14], [16], [22], and their representative strains were specifically annotated after the strain names. The phylogenetic trees were constructed by Maximum Likelihood using GARLI version 0.951 by selecting the GTR+I+G model from Modeltest 3.7. Trees were unrooted.
(3.38 MB PDF)

Figure S2 Phylogenetic tree of the HA gene of HK821-like avian influenza viruses isolated from Vietnam. HK821-like viruses formed three sub-lineages: HK821P, HK821α, and HK821β. The tree was rooted by Dk/HK/821/02. The phylogenetic tree was constructed by Maximum Likelihood using GARLI version 0.951 by selecting GTR+I+G model from Modeltest 3.7. Posterior probabilities and bootstrap values were given above and below branches, respectively.
(0.11 MB PDF)
