# Supplementary material for: Evolution of Highly Pathogenic H5N1 Avian Influenza Viruses in Vietnam between 2001 and 2007
Source: PLoS One. 2008 Oct 21;3(10):e3462. doi: 10.1371/journal.pone.0003462 (PMC2565130; doi:10.1371/journal.pone.0003462)
Supplement: Table S1 — Summary of the HPAI H5N1 viruses analyzed in this study. (0.86 MB PDF) [file pone.0003462.s001.pdf]

Table S1. Summary of the HPAI H5N1 viruses analyzed in this study.

Year	Virus	Source	Genotype	Location	Accession Number PB2	PB1	PA	HA	NP	NA	MP	NS	Segment Analysis PB2	PB1	PA	HA	NP	NA	MP	NS
2001	Gs/VN/113/01	Public	VN1					EF541399		EF541474	EF541393					GX22		F1		
2001	Gs/VN/324/01	Public	VN1					EF541400								GX22		F1		
2003	Ck/VN/19/03	Public	VN3	HA TAY	DQ492854	DQ493378	DQ493291	DQ497678	DQ493116	DQ493027	DQ492939	DQ493203	HK821	HK821	HK821	HK821	HK821	HK821	HK821	HK821
2003	Ck/VN/20/03	Public	VN3	HA TAY	DQ492855	DQ493379	DQ493292	DQ497679	DQ493117	DQ493028	DQ492940	DQ493204	HK821	HK821	HK821	HK821	HK821	HK821	HK821	HK821
2003	Ck/VN/27/03	Public	VN3	HA TAY	DQ320872	DQ321332	DQ321266	DQ320938	DQ321134	DQ321069	DQ321003	DQ321200	HK821	HK821	HK821	HK821	HK821	HK821	HK821	HK821
2003	Ck/VN/28/03	Public	VN3	HA TAY	DQ492857	DQ493381	DQ493294	DQ497681	DQ493119	DQ493030	DQ492942	DQ493206	HK821	HK821	HK821	HK821	HK821	HK821	HK821	HK821
2003	Ck/VN/30/03	Public	VN3	HA TAY	DQ492858	DQ493382	DQ493295	DQ497682	DQ493120	DQ493031	DQ492943	DQ493207	HK821	HK821	HK821	HK821	HK821	HK821	HK821	HK821
2003	Ck/VN/4/03	Public	VN3	VINH PHUC	DQ492867	DQ493391	DQ493304	DQ497691	DQ493129	DQ493040	DQ492952	DQ493216	HK821	HK821	HK821	HK821	HK821	HK821	HK821	HK821
2003	Ck/VN/5/03	Public	VN3	VINH PHUC	DQ492868	DQ493392	DQ493305	DQ497692	DQ493130	DQ493041	DQ492953	DQ493217	HK821	HK821	HK821	HK821	HK821	HK821	HK821	HK821
2003	Ck/VN/8/03	Public	VN3	VINH PHUC	DQ492869	DQ493393	DQ493306	DQ497693	DQ493131	DQ493042	DQ492954	DQ493218	HK821	HK821	HK821	HK821	HK821	HK821	HK821	HK821
2003	Dk/VN/15/03	Public	VN3	HA NOI	DQ492846	DQ493370	DQ493285	DQ497670	DQ493108	DQ493019	DQ492931	DQ493195	HK821	HK821	HK821	HK821	HK821	HK821	HK821	HK821
2003	Dk/VN/17/03	Public	VN3	HA NOI	DQ492848	DQ493372	DQ493286	DQ497672	DQ493110	DQ493021	DQ492933	DQ493197	HK821	HK821	HK821	HK821	HK821	HK821	HK821	HK821
2003	mallard/VN/16/03	Public	VN3	HA NOI	DQ492847	DQ493371	DQ493284	DQ497671	DQ493109	DQ493020	DQ492932	DQ493196	HK821	HK821	HK821	HK821	HK821	HK821	HK821	HK821
2003	mallard/VN/21/03	Public	VN3	HA TAY	DQ492856	DQ493380	DQ493293	DQ497680	DQ493118	DQ493029	DQ492941	DQ493205	HK821	HK821	HK821	HK821	HK821	HK821	HK821	HK821
2003	mallard/VN/3/03	Public	VN3	VINH PHUC	DQ492866	DQ493390	DQ493303	DQ497690	DQ493128	DQ493039	DQ492951	DQ493215	HK821	HK821	HK821	HK821	HK821	HK821	HK821	HK821
2003	Ck/VN/NCVD10/03	NCVD		VINH PHUC				ISDN40328	ISDN40077	ISDN48806	ISDN39969	ISDN40025				HK821	HK821	HK821	HK821	HK821
2003	Ck/VN/NCVD11/03	NCVD		VINH PHUC				ISDN40329		ISDN48807	ISDN39970	ISDN40026				HK821		HK821	HK821	HK821
2003	Ck/VN/NCVD13/03	NCVD		PHU THO	ISDN40368	ISDN40850	ISDN40951	ISDN40331	ISDN40079	ISDN38707		ISDN40028	F1(GX4016)	F1	F1	F1	F1	F1		F1(GX4016)
2003	Ck/VN/NCVD15/03	NCVD		VINH PHUC	ISDN40370	ISDN40852		ISDN40333	ISDN40081	ISDN38708	ISDN39973	ISDN40030	HK821	HK821		HK821	HK821	HK821	HK821	HK821
2003	Ck/VN/NCVD16/03	NCVD		BAC NINH				ISDN40912		ISDN48809	ISDN39974	ISDN40031				HK821		HK821	HK821	HK821
2003	Ck/VN/NCVD3/03	NCVD		HA NOI	ISDN40365			ISDN40322	ISDN40071	ISDN48801	ISDN39962	ISDN40019	F1(GX4016)			F1	F1	F1	E319(GX4016	F1(GX4016)
2003	Ck/VN/NCVD30/03	NCVD	VN3		ISDN40376	ISDN40857	ISDN40938	ISDN40916	ISDN40085	ISDN38712	ISDN39982	ISDN40039	HK821	HK821	HK821	HK821	HK821	HK821	HK821	HK821
2003	Ck/VN/NCVD4/03	NCVD		HA NOI				ISDN40323	ISDN40072	ISDN48802	ISDN39963	ISDN40020				F1	F1	F1	E319(GX4016	F1(GX4016)
2003	Ck/VN/NCVD5/03	NCVD		HA NOI				ISDN40324	ISDN40073	ISDN48803	ISDN39964	ISDN40021				F1	F1	F1	E319(GX4016	F1(GX4016)
2003	Ck/VN/NCVD6/03	NCVD		VINH PHUC				ISDN40325	ISDN40074	ISDN48804	ISDN39965	ISDN40022				HK821	HK821	HK821	HK821	HK821
2003	Ck/VN/NCVD7/03	NCVD	VN3	VINH PHUC	ISDN40366	ISDN40848	ISDN40949	ISDN38690	ISDN40075	ISDN38698	ISDN39966	ISDN40023	HK821	HK821	HK821	HK821	HK821	HK821	HK821	HK821
2003	Ck/VN/NCVD8/03	NCVD	VN2	HA NOI	ISDN40367	ISDN40849	ISDN40948	EF541407	ISDN45792	EF541468	EF541455	ISDN45786	F1(GX4016)	F1	F1	F1	F1	F1	E319(GX4016	F1(GX4016)
2003	Dk/VN/NCVD1/03	NCVD		HA TAY	ISDN40363	ISDN40846		EF541405	ISDN40070	EF541465	EF541454	ISDN40018	F1(GX4016)	F1		F1	F1	F1	E319(GX4016	F1(GX4016)
2003	Dk/VN/NCVD19/03	NCVD		HA TAY				ISDN40336	ISDN41052	ISDN48812	ISDN39977	ISDN40034				HK821	HK821	HK821	HK821	HK821
2003	Dk/VN/NCVD22/03	NCVD		HUNG YEN				ISDN40338	ISDN41054	ISDN48814	ISDN45750	ISDN45782				HK821	HK821	HK821	HK821	
2003	Dk/VN/NCVD24/03	NCVD		HUNG YEN				ISDN40389	ISDN41055	ISDN48815	ISDN39981	ISDN41015				HK821	HK821	HK821	HK821	HK821
2003	Dk/VN/NCVD25/03	NCVD	VN2		ISDN40373	ISDN40854	ISDN40947	ISDN40339	ISDN45790	ISDN38710	ISDN45805	ISDN45783	F1(GX4016)	F1	F1	F1	F1	F1	E319(GX4016	F1(GX4016)
2003	Dk/VN/NCVD29/03	NCVD	VN3	HA TAY	ISDN40375	ISDN40856	ISDN40946	ISDN40340	ISDN45791	ISDN38711	ISDN45807	ISDN45784	HK821	HK821	HK821	HK821	HK821	HK821	HK821	HK821
2003	M.Dk/VN/NCVD12/03	NCVD		HA TAY				ISDN40330	ISDN40078	ISDN48808	ISDN39971	ISDN40027				F1	F1	F1	E319(GX4016	F1(GX4016)
2003	M.Dk/VN/NCVD14/03	NCVD	VN3	VINH PHUC	ISDN40369	ISDN40851	ISDN40944	ISDN40332	ISDN40080	ISDN38699	ISDN39972	ISDN40029	HK821	HK821	HK821	HK821	HK821	HK821	HK821	HK821
2003	M.Dk/VN/NCVD17/03	NCVD		HA TAY				ISDN40334	ISDN41050	ISDN48810	ISDN39975	ISDN40032				HK821	HK821	HK821	HK821	HK821
2003	M.Dk/VN/NCVD18/03	NCVD		HA TAY				ISDN40335	ISDN41051	ISDN48811	ISDN39976	ISDN40033				HK821	HK821	HK821	HK821	HK821

2003	M.Dk/VN/NCVD2/03	NCVD		HA TAY	ISDN40364	ISDN40847		ISDN49462		ISDN48800	ISDN39961		F1(GX4016)	F1		F1		F1	E319(GX4016)	
2003	M.Dk/VN/NCVD20/03	NCVD		HA TAY				ISDN40337	ISDN41053	ISDN48813	ISDN39978	ISDN40035				HK821	HK821	HK821	HK821	HK821
2003	M.Dk/VN/NCVD21/03	NCVD	VN3	HUNG YEN	ISDN40371	ISDN40853	ISDN40942	ISDN38691	ISDN40082	ISDN38700	ISDN39979	ISDN40036	HK821	HK821	HK821	HK821	HK821	HK821	HK821	HK821
2003	M.Dk/VN/NCVD23/03	NCVD		BAC NINH	ISDN40372			ISDN40915	ISDN40083	ISDN38709	ISDN39980	ISDN40037	HK821			HK821	HK821	HK821	HK821	HK821
2003	M.Dk/VN/NCVD28/03	NCVD	VN3	HA TAY	ISDN40374	ISDN40855	ISDN40941	ISDN40913	ISDN40084	ISDN38701	ISDN45806	ISDN40038	HK821	HK821	HK821	HK821	HK821	HK821	HK821	HK821
2003	M.Dk/VN/NCVD9/03	NCVD		HA NOI				ISDN40327	ISDN40076	ISDN48805	ISDN39968	ISDN40024				F1	F1	F1	E319(GX4016	F1(GX4016)
2004	Ck/VN/G04/04	Public						ISDN128306		ISDN128352						HK821		HK821		
2004	Ck/VN/1/04	Public						EF541409		EF541475	EF541449		HK821	HK821	HK821	HK821		HK821	HK821	HK821
2004	Ck/VN/132/04	Public	VN3	VINH LONG	DQ492892	DQ493417	DQ493331	DQ497718	DQ493155	DQ493067	DQ492979	DQ493243	HK821	HK821	HK821	HK821	HK821	HK821	HK821	HK821
2004	Ck/VN/133/04	Public	VN3	CAN THO	DQ492875	DQ493399	DQ493313	DQ497700	DQ493138	DQ493049	DQ492961	DQ493225	HK821	HK821	HK821	HK821	HK821	HK821	HK821	HK821
2004	Ck/VN/134/04	Public		TIEN GIANG	DQ492889	DQ493413	DQ493327	DQ497714	DQ493151	DQ493063	DQ492975	DQ493239	HK821	HK821	Not defined	HK821	HK821	HK821	HK821	HK821
2004	Ck/VN/135/04	Public	VN3	BAC LIEU	DQ492871	DQ493395	DQ493308	DQ497695	DQ493133	DQ493044	DQ492956	DQ493220	HK821	HK821	HK821	HK821	HK821	HK821	HK821	HK821
2004	Ck/VN/147/04	Public	VN3	CAN THO	DQ492876	DQ493400	DQ493314	DQ497701	DQ493139	DQ493050	DQ492962	DQ493226	HK821	HK821	HK821	HK821	HK821	HK821	HK821	HK821
2004	Ck/VN/149/04	Public	VN3	BAC LIEU	DQ492872	DQ493396	DQ493309	DQ497696	DQ493134	DQ493045	DQ492957	DQ493221	HK821	HK821	HK821	HK821	HK821	HK821	HK821	HK821
2004	Ck/VN/159/04	Public	VN3	SOC TRANG	DQ492888	DQ493412	DQ493326	DQ497713	DQ493150	DQ493062	DQ492974	DQ493238	HK821	HK821	HK821	HK821	HK821	HK821	HK821	HK821
2004	Ck/VN/260/04	Public	VN3	HAU GIANG	DQ492881	DQ493405	DQ493319	DQ497706	DQ493144	DQ493055	DQ492967	DQ493231	HK821	HK821	HK821	HK821	HK821	HK821	HK821	HK821
2004	Ck/VN/32/04	Public	VN3	THAI BINH	DQ492863	DQ493387	DQ493300	DQ497687	DQ493125	DQ493036	DQ492948	DQ493212	HK821	HK821	HK821	HK821	HK821	HK821	HK821	HK821
2004	Ck/VN/33/04	Public	VN3		AY651722	AY651668	AY651614	AY651337	AY651502	AY651449	AY651391	AY651556	HK821	HK821	HK821	HK821	HK821	HK821	HK821	HK821
2004	Ck/VN/35/04	Public	VN3		AY651723	AY651669	AY651615	AY651338	AY651503	AY651450	AY651392	AY651557	HK821	HK821	HK821	HK821	HK821	HK821	HK821	HK821
2004	Ck/VN/36/04	Public	VN3		AY651724	AY651670	AY651616	AY651339	AY651504	AY651451	AY651393	AY651558	HK821	HK821	HK821	HK821	HK821	HK821	HK821	HK821
2004	Ck/VN/37/04	Public	VN3		AY651725	AY651671	AY651617	AY651340	AY651505	AY651452	AY651394	AY651559	HK821	HK821	HK821	HK821	HK821	HK821	HK821	HK821
2004	Ck/VN/38/04	Public	VN3		AY651726	AY651672	AY651618	AY651341	AY651506	AY651453	AY651395	AY651560	HK821	HK821	HK821	HK821	HK821	HK821	HK821	HK821
2004	Ck/VN/39/04	Public	VN3		AY651727	AY651673	AY651619	AY651342	AY651507	AY651454	AY651396	AY651561	HK821	HK821	HK821	HK821	HK821	HK821	HK821	HK821
2004	Ck/VN/486A/04	Public								DQ884460								HK821		
2004	Ck/VN/52/04	Public	VN3	BINH DINH	DQ492874	DQ493398	DQ493311	DQ497698	DQ493136	DQ493047	DQ492959	DQ493223	HK821	HK821	HK821	HK821	HK821	HK821	HK821	HK821
2004	Ck/VN/53/04	Public	VN3	KHANH HOA	DQ492885	DQ493409	DQ493323	DQ497710	DQ493147	DQ493059	DQ492971	DQ493235	HK821	HK821	HK821	HK821	HK821	HK821	HK821	HK821
2004	Ck/VN/AG-010/04	Public	VN3	AN GIANG	DQ138170	DQ138149	AY724790	AY724789	DQ099765	DQ094278	DQ094256	AY770612	HK821	HK821	HK821	HK821	HK821	HK821	HK821	HK821
2004	Ck/VN/C57/04	Public	VN3		AY651728	AY651674	AY651620	AY651343	AY651508	AY651455	AY651397	AY651562	HK821	HK821	HK821	HK821	HK821	HK821	HK821	HK821
2004	Ck/VN/C58/04	Public	VN3		AY818127	AY818130	AY818133	AY818136	AY818139	AY818142	AY818145	AY818148	HK821	HK821	HK821	HK821	HK821	HK821	HK821	HK821
2004	Ck/VN/CM/04	Public		CA MAU				AY576927	AY576929	AY576928						HK821	HK821	HK821		
2004	Ck/VN/CT-018/04	Public	VN3	CAN THO	DQ138172	DQ138151	DQ099786	AY724793	DQ099767	DQ094280	DQ094258	AY770614	HK821	HK821	HK821	HK821	HK821	HK821	HK821	HK821
2004	Ck/VN/DN-045/04	Public	VN3	DONG NAI	DQ138176	DQ138155	AY724786	AY724785	DQ099771	DQ094284	DQ094262	AY770618	HK821	HK821	HK821	HK821	HK821	HK821	HK821	HK821
2004	Ck/VN/DT-015/04	Public	VN3	DONG THAP	DQ138171	DQ138150	AY724792	AY724791	DQ099766	DQ094279	DQ094257	AY770613	HK821	HK821	HK821	HK821	HK821	HK821	HK821	HK821
2004	Ck/VN/DT171/04	Public	VN3	DONG THAP	DQ320871	DQ321331	DQ321265	DQ320937	DQ321133	DQ321068	DQ321006	DQ321199	HK821	HK821	HK821	HK821	HK821	HK821	HK821	HK821
2004	Ck/VN/DT-171/04	Public	VN3	DONG THAP	DQ138177	DQ138156	DQ099788	DQ099759	DQ099772	AY720943	DQ094263	AY770619	HK821	HK821	HK821	HK821	HK821	HK821	HK821	HK821
2004	Ck/VN/HauGiang-178/04	Public		HAU GIANG				AY728892		AY728893						HK821		HK821		
2004	Ck/VN/HauGiang-617/04	Public		HAU GIANG				AY728894		AY728895						HK821		HK821		
2004	Ck/VN/HCM-022/04	Public	VN3	HOU CHI MING	DQ138175	DQ138154	AY724784	AY724783	DQ099770	DQ094283	DQ094261	AY770617	HK821	HK821	HK821	HK821	HK821	HK821	HK821	HK821
2004	Ck/VN/HD1/04	Public		HA TAY				AY574187	AY574189	AY574188						HK821	HK821	HK821		
2004	Ck/VN/HD2/04	Public		HA TAY				AY574190	AY574192	AY574191						HK821	HK821	HK821		
2004	Ck/VN/KG-076/04	Public		KIEN GIANG		DQ138157	DQ099789		DQ099773	DQ094285	DQ094264	AY770620		HK821	HK821		HK821	HK821	HK821	HK821

2004	Ck/VN/LA-024/04	Public	VN3	LONG AN	DQ138174	DQ138153	AY724796	AY724795	DQ099769	DQ094282	DQ094260	AY770616	HK821	HK821	HK821	HK821	HK821	HK821	HK821	HK821
2004	Ck/VN/LD-080/04	Public	VN3	LAM DONG	DQ138178	DQ138158	DQ099790	DQ099760	DQ099774	DQ094287	DQ094265	AY770621	HK821	HK821	HK821	HK821	HK821	HK821	HK821	HK821
2004	Ck/VN/TG-023/04	Public	VN3	TIEN GIANG	DQ138173	DQ138152	DQ099787	DQ099758	DQ099768	DQ094281	DQ094259	AY770615	HK821	HK821	HK821	HK821	HK821	HK821	HK821	HK821
2004	Ck/VN/TN-025/04	Public	VN3	TAY NINH	DQ138166	DQ138145	DQ099784	DQ099755	DQ099761	DQ094276	DQ094252	AY770609	HK821	HK821	HK821	HK821	HK821	HK821	HK821	HK821
2004	Ck/VN/VL-008/04	Public	VN3	VINH LONG	DQ138169	DQ138148	AY724788	AY724787	DQ099764	DQ094277	DQ094255	AY770611	HK821	HK821	HK821	HK821	HK821	HK821	HK821	HK821
2004	Dk/VN/11/04	Public	VN3		AY651729	AY651675	AY651621	AY651344	AY651509	AY651456	AY651398	AY651563	HK821	HK821	HK821	HK821	HK821	HK821	HK821	HK821
2004	Dk/VN/148/04	Public	VN3	CAN THO	DQ492877	DQ493401	DQ493315	DQ497702	DQ493140	DQ493051	DQ492963	DQ493227	HK821	HK821	HK821	HK821	HK821	HK821	HK821	HK821
2004	Dk/VN/219/04	Public	VN3	HA TAY	DQ492860	DQ493384	DQ493297	DQ497684	DQ493122	DQ493033	DQ492945	DQ493209	HK821	HK821	HK821	HK821	HK821	HK821	HK821	HK821
2004	Dk/VN/220/04	Public	VN3	HA TAY	DQ492861	DQ493385	DQ493298	DQ497685	DQ493123	DQ493034	DQ492946	DQ493210	HK821	HK821	HK821	HK821	HK821	HK821	HK821	HK821
2004	Dk/VN/258/04	Public	VN3	TRA VINH	DQ492893	DQ493415	DQ493329	DQ497716	DQ493153	DQ493065	DQ492977	DQ493241	HK821	HK821	HK821	HK821	HK821	HK821	HK821	HK821
2004	Dk/VN/40/04	Public	VN3	HA NOI	DQ492849	DQ493373	DQ493287	DQ497673	DQ493111	DQ493022	DQ492934	DQ493198	HK821	HK821	HK821	HK821	HK821	HK821	HK821	HK821
2004	Dk/VN/48/04	Public	VN3	HA TAY	DQ492859	DQ493383	DQ493296	DQ497683	DQ493121	DQ493032	DQ492944	DQ493208	HK821	HK821	HK821	HK821	HK821	HK821	HK821	HK821
2004	Dk/VN/CM-V7/04	Public		CA MAU	DQ138180	DQ138162	DQ138183		DQ099778	DQ094289	DQ094269		HK821	HK821	HK821		HK821	HK821	HK821	
2004	Dk/VN/N-XX/04	Public	VN3	HA TAY	DQ492862	DQ493386	DQ493299	DQ497686	DQ493124	DQ493035	DQ492947	DQ493211	HK821	HK821	HK821	HK821	HK821	HK821	HK821	HK821
2004	Dk/VN/TG-007A/04	Public	VN3	TIEN GIANG	DQ138167	DQ138146	AY720947	DQ099756	DQ099762	AY720946	DQ094253	AY770610	HK821	HK821	HK821	HK821	HK821	HK821	HK821	HK821
2004	Dk/VN/TV-V2/04	Public		TRA VINH	DQ138179	DQ138161	DQ138182		DQ099777	DQ094288	DQ094268		HK821	HK821	HK821		HK821	HK821	HK821	
2004	Gs/VN/264/04	Public	VN3	HAU GIANG	DQ492882	DQ493406	DQ493320	DQ497707	DQ493145	DQ493056	DQ492968	DQ493232	HK821	HK821	HK821	HK821	HK821	HK821	HK821	HK821
2004	Hanoi/03/04	Public		HA NOI				AJ715872	AJ715873							HK821	HK821			
2004	Hatay/04/	Public		HA TAY				AJ867074	AJ867076	AJ867075	AM040045	AM040046					HK821	HK821	HK821	HK821
2004	M.Dk/VN/MdGL/04	Public						AY576930	AY576931							HK821	HK821			
2004	MallardDk/VN/133/04	Public			DQ320873			DQ320940					HK821			HK821				
2004	quail/VN/177/04	Public	VN3	TRA VINH	DQ492890	DQ493414	DQ493328	DQ497715	DQ493152	DQ493064	DQ492976	DQ493240	HK821	HK821	HK821	HK821	HK821	HK821	HK821	HK821
2004	quail/VN/36/04	Public	VN3		AY818128	AY818131	AY818134	AY818137	AY818140	AY818143	AY818146	AY818149	HK821	HK821	HK821	HK821	HK821	HK821	HK821	HK821
2004	quail/VN/TG-007B/04	Public		TIEN GIANG	DQ138168	DQ138147	DQ099785	DQ099757	DQ099763		DQ094254		HK821	HK821	HK821	HK821	HK821		HK821	
2004	VN/1194/04	Public	VN3		AY651718	AY651664	AY651610	EF541402	AY651498	EF541466	AY651387	AY651552	HK821	HK821	HK821	HK821	HK821	HK821	HK821	HK821
2004	VN/1196/04	Public	VN3		AY526752	AY526751	AY526750	AY526745	AY526749	AY526746	AY526748	AY526747	HK821	HK821	HK821	HK821	HK821	HK821	HK821	HK821
2004	VN/1203/04	Public	VN3		EF467805	EF467808	AY818132	EF541403	AY818138	EF541467	AY818144	AY818147	HK821	HK821	HK821	HK821	HK821	HK821	HK821	HK821
2004	VN/1204/04	Public			EF467806	EF467809	EF473407	EF541404				EF541457	HK821	HK821	HK821	HK821				HK821
2004	VN/3046/04	Public	VN3		AY651720	AY651666	AY651613	AY651335	AY651500	AY651446	AY651389	AY651554	HK821	HK821	HK821	HK821	HK821	HK821	HK821	HK821
2004	VN/3062/04	Public	VN3		AY651721	AY651667	AY651612	AY651336	AY651501	AY651448	AY651390	AY651555	HK821	HK821	HK821	HK821	HK821	HK821	HK821	HK821
2004	VN/3212/04	Public						EF451059					HK821	HK821	HK821	HK821		HK821	HK821	HK821
2004	VN/33/04	Public							DQ099776								HK821			
2004	VN/CL01/04	Public	VN3	HOU CHI MING	DQ492894	DQ493418	DQ493332	DQ497719	DQ493156	DQ250159	DQ492980	DQ493244	HK821	HK821	HK821	HK821	HK821	HK821	HK821	HK821
2004	VN/CL02/04	Public		SOC TRANG	DQ492895	DQ493419		DQ497720	DQ493157	DQ493069	DQ492981	DQ493245	HK821	HK821		HK821	HK821	HK821	HK821	HK821
2004	VN/CL100/04	Public	VN3	TAY NINH	DQ492898	DQ493424	DQ493337	DQ497725	DQ493162	DQ250162	DQ492986	DQ493250	HK821	HK821	HK821	HK821	HK821	HK821	HK821	HK821
2004	VN/CL17/04	Public		LAM DONG		DQ493420	DQ493333	DQ497721	DQ493158	DQ493070	DQ492982	DQ493246		HK821	HK821		HK821	HK821	HK821	HK821
2004	VN/CL20/04	Public		LAM DONG		DQ493421	DQ493334	DQ497722	DQ493159	DQ493071	DQ492983	DQ493247		HK821	HK821		HK821	HK821	HK821	HK821
2004	VN/CL26/04	Public	VN3	LAM DONG	DQ492896	DQ493422	DQ493335	DQ497723	DQ493160	DQ493072	DQ492984	DQ493248	HK821	HK821	HK821	HK821	HK821	HK821	HK821	HK821
2004	VN/CL36/04	Public	VN3	BINH DINH	DQ492897	DQ493423	DQ493336	DQ497724	DQ493161	DQ250161	DQ492985	DQ493249	HK821	HK821	HK821	HK821	HK821	HK821	HK821	HK821
2004	VN/DN-33/04	Public		DONG NAI		DQ138159	DQ099791	AY720950		AY720948	DQ094266	AY720949		HK821	HK821	HK821		HK821	HK821	HK821
2004	VN/HG-178/04	Public		HA GIANG		DQ138160	DQ099792		DQ099775	DQ094286	DQ094267			HK821	HK821		HK821	HK821	HK821	

2004	VN/HN/04	Public		HA NOI	AY720954	AY720955	AY720952		AY720953		AY720951		HK821	HK821	HK821		HK821		HK821	
2004	VN/JP178/04	Public		HA NOI				EF456795		EF456796		ISDN69612	HK821	HK821	HK821	HK821	HK821	HK821	HK821	HK821
2004	Ck/VN/NCVD31/04	NCVD	VN3	HA TAY	ISDN40377	ISDN40858	ISDN40950	EF541406	ISDN41056	ISDN38702	ISDN45754	ISDN45785	HK821	HK821	HK821	HK821	HK821	HK821	HK821	HK821
2005	Ck/VN/10/05	Public	VN4		CY016874	CY016873	CY016872	CY016867	CY016870	CY016869	CY016868	CY016871	F1(GX4016)	GX4016	GX4016	E319(GX401	E319(FJ584)((	GX4016(E319)	E319(GX4016	F1(GX4016)
2005	Ck/VN/11/05	Public	VN3		CY016882	CY016881	CY016880	CY016875	CY016878	CY016877	CY016876	CY016879	HK821	HK821	HK821	HK821	HK821	HK821	HK821	HK821
2005	Ck/VN/2/05	Public	VN3		CY016842	CY016841	CY016840	CY016835	CY016838	CY016837	CY016836	CY016839	HK821	HK821	HK821	HK821	HK821	HK821	HK821	HK821
2005	Ck/VN/348/05	Public	VN3	HA NOI	DQ492852	DQ493376	DQ493289	DQ497676	DQ493114	DQ493025	DQ492937	DQ493201	HK821	HK821	HK821	HK821	HK821	HK821	HK821	HK821
2005	Ck/VN/393/05	Public	VN3	KIEN GIANG	DQ492887	DQ493411	DQ493325	DQ497712	DQ493149	DQ493061	DQ492973	DQ493237	HK821	HK821	HK821	HK821	HK821	HK821	HK821	HK821
2005	Ck/VN/398/05	Public	VN3	DONG THAP	DQ492878	DQ493402	DQ493316	DQ497703	DQ493141	DQ493052	DQ492964	DQ493228	HK821	HK821	HK821	HK821	HK821	HK821	HK821	HK821
2005	Ck/VN/6/05	Public	VN3		CY016850	CY016849	CY016848	CY016843	CY016846	CY016845	CY016844	CY016847	HK821	HK821	HK821	HK821	HK821	HK821	HK821	HK821
2005	Ck/VN/8/05	Public	VN3		CY016858	CY016857	CY016856	CY016851	CY016854	CY016853	CY016852	CY016855	HK821	HK821	HK821	HK821	HK821	HK821	HK821	HK821
2005	Ck/VN/9/05	Public	VN3		CY016866	CY016865	CY016864	CY016859	CY016862	CY016861	CY016860	CY016863	HK821	HK821	HK821	HK821	HK821	HK821	HK821	HK821
2005	Ck/VN/TY25/05	Public		THAI NGUYEN				ISDN128309		ISDN128355									HK821	
2005	Ck/VN/TY31/05	Public		THAI NGUYEN				ISDN128310		ISDN128356						E319(GX4016)(GX604)		GX4016(E319)		
2005	Ck/VN/TY9/05	Public		THAI NGUYEN				ISDN128308		ISDN128354						HK821		HK821		
2005	Ck/VN/CT-437/05	Public									DQ094270								HK821	
2005	Ck/VN/P22/05	Public						AM183674		AM183679						HK821		HK821		
2005	Ck/VN/P41/05	Public						AM183672								HK821				
2005	Ck/VN/P78/05	Public						AM183673								HK821				
2005	Dk/VN/1/05	Public	VN5		CY016834	CY016833	CY016832	CY016827	CY016830	CY016829	CY016828	CY016831	HK97	HK97	HK97	HK97	HK97	HK97	HK97	HK97
2005	Dk/VN/1/05a	Public	VN3		DQ366303	DQ366304	DQ366305	DQ366306	DQ366307	DQ366308	DQ366309	DQ366310	HK821	HK821	HK821	HK821	HK821	HK821	HK821	HK821
2005	Dk/VN/12/05	Public	VN4		CY016890	CY016889	CY016888	CY016883	CY016886	CY016885	CY016884	CY016887	F1(GX4016)	GX4016	GX4016	E319(GX401	E319(FJ584)((	GX4016(E319)	E319(GX4016	F1(GX4016)
2005	Dk/VN/18/05	Public	VN3		CY017074	CY017073	CY017072	CY017067	CY017070	CY017069	CY017068	CY017071	HK821	HK821	HK821	HK821	HK821	HK821	HK821	HK821
2005	Dk/VN/19/05	Public	VN3		CY017194	CY017193	CY017192	CY017187	CY017190	CY017189	CY017188	CY017191	HK821	HK821	HK821	HK821	HK821	HK821	HK821	HK821
2005	Dk/VN/20/05	Public	VN3		CY016898	CY016897	CY016896	CY016891	CY016894	CY016893	CY016892	CY016895	HK821	HK821	HK821	HK821	HK821	HK821	HK821	HK821
2005	Dk/VN/272/05	Public	VN3	HA NOI	DQ492850	DQ493374	DQ493288	DQ497674	DQ493112	DQ493023	DQ492935	DQ493199	HK821	HK821	HK821	HK821	HK821	HK821	HK821	HK821
2005	Dk/VN/283/05	Public	VN3	HAU GIANG	DQ492883	DQ493407	DQ493321	DQ497708	DQ493107	DQ493057	DQ492969	DQ493233	HK821	HK821	HK821	HK821	HK821	HK821	HK821	HK821
2005	Dk/VN/286/05	Public	VN3	BAC LIEU	DQ492873	DQ493397	DQ493310	DQ497697	DQ493135	DQ493046	DQ492958	DQ493222	HK821	HK821	HK821	HK821	HK821	HK821	HK821	HK821
2005	Dk/VN/317/05	Public	VN3	THAI BINH	DQ492865	DQ493389	DQ493302	DQ497689	DQ493127	DQ493038	DQ492950	DQ493214	HK821	HK821	HK821	HK821	HK821	HK821	HK821	HK821
2005	Dk/VN/367/05	Public		DONG THAP				EF566215		EF566216						HK821		HK821		
2005	Dk/VN/376/05	Public	VN3	TRA VINH	DQ492879	DQ493403	DQ493317	DQ497704	DQ493142	DQ493053	DQ492965	DQ493229	HK821	HK821	HK821	HK821	HK821	HK821	HK821	HK821
2005	Dk/VN/543/05	Public	VN3	HAU GIANG	DQ492891	DQ493416	DQ493330	DQ497717	DQ493154	DQ493066	DQ492978	DQ493242	HK821	HK821	HK821	HK821	HK821	HK821	HK821	HK821
2005	Dk/VN/557/05	Public	VN3		DQ492884	DQ493408	DQ493322	DQ497709	DQ493146	DQ493058	DQ492970	DQ493234	HK821	HK821	HK821	HK821	HK821	HK821	HK821	HK821
2005	Dk/VN/568/05	Public	VN4		DQ320874	DQ321333	DQ321267	DQ320939	DQ321135	DQ321070	DQ321005	DQ321201	F1(GX4016)	GX4016	GX4016	E319(GX401	E319(FJ584)((	GX4016(E319)	E319(GX4016	F1(GX4016)
2005	Dk/VN/8/05	Public	VN5		DQ366319	DQ366320	DQ366321	DQ366322	DQ366323	DQ366324	DQ366325	DQ366326	HK97	HK97	HK97	HK97	HK97	HK97	HK97	HK97
2005	Dk/VN/AG40-O2/05	Public		AN GIANG				AM183676								HK821				
2005	Dk/VN/N-TB/05	Public	VN3	THAI BINH	DQ492864	DQ493388	DQ493301	DQ497688	DQ493126	DQ493037	DQ492949	DQ493213	HK821	HK821	HK821	HK821	HK821	HK821	HK821	HK821
2005	Dk/VN/S640/05	Public	VN3	AN GIANG	DQ492870	DQ493394	DQ493307	DQ497694	DQ493132	DQ493043	DQ492955	DQ493219	HK821	HK821	HK821	HK821	HK821	HK821	HK821	HK821
2005	Dk/VN/S649/05	Public		CA MAU			DQ493312	DQ497699	DQ493137	DQ493048	DQ492960	DQ493224			HK821	HK821	HK821	HK821	HK821	HK821
2005	Dk/VN/S654/05	Public	VN3	CAN THO	DQ320863	DQ321335	DQ321269	DQ320936	DQ321137	DQ321067	DQ321002	DQ321203	HK821	HK821	HK821	HK821	HK821	HK821	HK821	HK821
2005	Dk/VN/TG24-O1/05	Public		TUYEN QUANG				AM183677		AM183678						HK821		HK821		

2005	Dk/VN/TG36-H2/05	Public		TUYEN QUANG				AM183675								HK821				
2005	Gs/VN/3/05	Public	VN5		DQ366311	DQ366312	DQ366313	DQ366314	DQ366315	DQ366316	DQ366317	DQ366318	HK97	HK97	HK97	HK97	HK97	HK97	HK97	HK97
2005	Hanoi/30408/05	Public		HA NOI				AB239125		AB239126						HK821		HK821		
2005	mallard/VN/347/05	Public	VN3	HA NOI	DQ492851	DQ493375	DQ493283	DQ497675	DQ493113	DQ493024	DQ492936	DQ493200	HK821	HK821	HK821	HK821	HK821	HK821	HK821	HK821
2005	mallard/VN/352/05	Public	VN3	HA NOI	DQ492853	DQ493377	DQ493290	DQ497677	DQ493115	DQ493026	DQ492938	DQ493202	HK821	HK821	HK821	HK821	HK821	HK821	HK821	HK821
2005	MallardDk/VN/133/05	Public		CAN THO		DQ321334	DQ321268		DQ321136	DQ321071	DQ321004	DQ321202		HK821	HK821		HK821	HK821	HK821	HK821
2005	Owston'scivet/VN/1/05	Public	VN4		EF124801	EF124800	EF124799	EF124794	EF124797	EF124796	EF124795	EF124798	F1(GX4016)	GX4016	GX4016	E319(GX401	E319(FJ584)(	GX4016(E319)	E319(GX4016	F1(GX4016)
2005	quail/VN/15/05	Public	VN4		CY017058	CY017057	CY017056	CY017051	CY017054	CY017053	CY017052	CY017055	F1(GX4016)	GX4016	GX4016	E319(GX401	E319(FJ584)(	GX4016(E319)	E319(GX4016	F1(GX4016)
2005	quail/VN/282/05	Public	VN3	KIEN GIANG	DQ492886	DQ493410	DQ493324	DQ497711	DQ493148	DQ493060	DQ492972	DQ493236	HK821	HK821	HK821	HK821	HK821	HK821	HK821	HK821
2005	VN/30850/05	Public	VN3		ISDN130364	ISDN130363	ISDN130362	ISDN130357	ISDN130361	ISDN130358	ISDN130359	ISDN130360	HK821	HK821	HK821	HK821	HK821	HK821	HK821	HK821
2005	VN/BL-014/05	Public		BAC LIEU		DQ138163	DQ138184		DQ099779	DQ094290	DQ094271			HK821	HK821		HK821	HK821	HK821	
2005	VN/CL105/05	Public	VN3	TRA VINH	DQ492899	DQ493425	DQ493338	DQ497726	DQ493163	DQ493075	DQ492987	DQ493251	HK821	HK821	HK821	HK821	HK821	HK821	HK821	HK821
2005	VN/CL115/05	Public	VN3	VINH LONG	DQ492900	DQ493426	DQ493339	DQ497727	DQ493164	DQ250163	DQ492988	DQ493252	HK821	HK821	HK821	HK821	HK821	HK821	HK821	HK821
2005	VN/CL119/05	Public	VN3	DONG THAP	DQ492901	DQ493427	DQ493340	DQ497728	DQ493165	DQ250164	DQ492989	DQ493253	HK821	HK821	HK821	HK821	HK821	HK821	HK821	HK821
2005	VN/CL2009/05	Public	VN3	DONG THAP	DQ492902	DQ493428	DQ493341	DQ497729	DQ493166	DQ250165	DQ492990	DQ493254	HK821	HK821	HK821	HK821	HK821	HK821	HK821	HK821
2005	VN/DT-036/05	Public		DONG THAP	DQ138181	DQ138164	DQ138185		DQ099780	DQ094291	DQ094272		HK821	HK821	HK821		HK821	HK821	HK821	
2005	VN/HG-207/05	Public		HA GIANG		DQ138165	DQ138186		DQ099781	DQ094292	DQ094273			HK821	HK821		HK821	HK821	HK821	
2005	VN/HN30408/05	Public		HA NOI				EF456803		EF456804						HK821		HK821		
2005	VN/JP14/05	Public						EF456799		EF456801						HK821		HK821		
2005	VN/JP4207/05	Public						EF456798		EF456800			HK821	HK821	HK821	HK821	HK821	HK821	HK821	HK821
2005	VN/JPHN30321/05	Public		HA NOI				EF456802					HK821	HK821	HK821	HK821	HK821	HK821	HK821	HK821
2005	VN/LA-028/05	Public		LONG AN			DQ138187		DQ099782		DQ094274				HK821		HK821		HK821	
2005	VN/PEV16T/05	Public	VN3		DQ535731	DQ535730	DQ535729	DQ535724	DQ535727	DQ535726	DQ535725	DQ535728	HK821	HK821	HK821	HK821	HK821	HK821	HK821	HK821
2005	VN/VL-020/05	Public		VINH LONG			DQ138188		DQ099783		DQ094275				HK821		HK821		HK821	
2005	wildbird/VN/434/05	Public	VN3	DONG THAP	DQ492880	DQ493404	DQ493318	DQ497705	DQ493143	DQ493054	DQ492966	DQ493230	HK821	HK821	HK821	HK821	HK821	HK821	HK821	HK821
2005	Ck/VN/LongAn636/05	Public		LONG AN				ISDN230180												
2005	Ck/VN/BinhDuong477/05	Public		BINH DUONG				ISDN230181												
2005	Dk/VN/DongThap680H/05	Public		DONG THAP				ISDN230182												
2005	Dk/VN/SocTrang680G/05	Public		SOC TRANG				ISDN230183												
2005	Dk/VN/HauGiang680F/05	Public		HAU GIANG				ISDN230184												
2005	Dk/VN/HauGiang680E/05	Public		HAU GIANG				ISDN230185												
2005	Dk/VN/DongThap680D/05	Public		DONG THAP				ISDN230186												
2005	Dk/VN/SocTrang680C/05	Public		SOC TRANG				ISDN230187												
2005	Dk/VN/AnGiang680B/05	Public		AN GIANG				ISDN230188												
2005	Dk/VN/DongThap680/05	Public		DONG THAP				ISDN230189												
2005	Dk/VN/5001/05	Public						ISDN128301		ISDN205311						HK821		HK821		
2005	Dk/VN/5003/05	Public						ISDN205303		ISDN205312						HK821		HK821		
2005	Dk/VN/5004/05	Public						ISDN205304		ISDN205313						HK821		HK821		
2005	Dk/VN/5082/05	Public						ISDN205305		ISDN205314						HK821		HK821		
2005	Ck/VN/TY25/05	Public						ISDN205310		ISDN205319						E319(GX4016)(GX604)		GX4016(E319)		

2005	Ck/VN/TY31/05	Public						ISDN205309		ISDN205318						E319(GX4016)(GX604)		GX4016(E319)		
2005	Ck/VN/TY9/05	Public						ISDN205308		ISDN205317						HK821		HK821		
2005	Ck/VN/G62/05	Public						ISDN205307		ISDN205316								HK821		
2005	Ck/VN/NCVD09/05	NCVD						EF566200		EF566212						HK821		HK821		
2005	Ck/VN/NCVD10/05	NCVD						EF566199		EF566213						HK821		HK821		
2005	Ck/VN/NCVD107/05	NCVD		NINH BINH				ISDN131241								E319(GX4016)(GX604)				
2005	Ck/VN/NCVD117/05	NCVD		HA TAY				ISDN131244								E319(GX4016)(GX604)				
2005	Ck/VN/NCVD12/05	NCVD						EF566198		EF566214						HK821		HK821		
2005	Ck/VN/NCVD142/05	NCVD		BAC NINH				ISDN131247								E319(GX4016)(GX604)				
2005	Ck/VN/NCVD143/05	NCVD		NAM DINH				ISDN131248								E319(GX4016)(GX604)				
2005	Ck/VN/NCVDCDC1/05	NCVD	VN3	HA TAY	ISDN131304	ISDN131249	ISDN125522	ISDN124063	ISDN125681	ISDN124098	ISDN125576	ISDN125624	HK821	HK821	HK821	HK821	HK821	HK821	HK821	HK821
2005	Ck/VN/NCVDCDC18/05	NCVD	VN3	HA NOI	ISDN131316	ISDN131264	ISDN125537	ISDN124076	ISDN125697	ISDN124111	ISDN125589	ISDN125641	HK821	HK821	HK821	HK821	HK821	HK821	HK821	HK821
2005	Ck/VN/NCVDCDC19/05	NCVD	VN3	HA NOI	ISDN131317	ISDN131265	ISDN125538	ISDN124077	ISDN125698	ISDN124112	ISDN125590	ISDN125642	HK821	HK821	HK821	HK821	HK821	HK821	HK821	HK821
2005	Ck/VN/NCVDCDC20/05	NCVD		HA NOI			ISDN131351					ISDN125643			HK821					HK821
2005	Ck/VN/NCVDCDC21/05	NCVD		HA NOI	ISDN131318	ISDN131266	ISDN131352					ISDN125644								
2005	Ck/VN/NCVDCDC22/05	NCVD	VN3	HA NOI	ISDN131319	ISDN131267	ISDN125539	ISDN124078	ISDN125699	ISDN124113	ISDN125591	ISDN125645	HK821	HK821	HK821	HK821	HK821	HK821	HK821	HK821
2005	Ck/VN/NCVDCDC24/05	NCVD	VN4	HAI DUONG	ISDN131320	ISDN131268	ISDN125540	ISDN124079	ISDN125700	ISDN124114	ISDN125592	ISDN125646	F1(GX4016)	GX4016	GX4016	E319(GX401	E319(FJ584)(	GX4016(E319)	E319(GX4016	F1(GX4016)
2005	Ck/VN/NCVDCDC25/05	NCVD	VN3	HAI DUONG	ISDN131321	ISDN131269	ISDN125541	ISDN124155	ISDN125701	ISDN124167	ISDN125593	ISDN125647	HK821	HK821	HK821	HK821	HK821	HK821	HK821	HK821
2005	Ck/VN/NCVDCDC26/05	NCVD		HAI DUONG	ISDN131322	ISDN131270	ISDN125542	ISDN124156	ISDN125702	ISDN124168		ISDN125648	HK821	HK821	HK821	HK821	HK821	HK821		HK821
2005	Ck/VN/NCVDCDC27/05	NCVD	VN3	BAC NINH	ISDN131323	ISDN131271	ISDN125543	ISDN124080	ISDN125703	ISDN124115	ISDN125594	ISDN125649	HK821	HK821	HK821	HK821	HK821	HK821	HK821	HK821
2005	Ck/VN/NCVDCDC28/05	NCVD		BAC NINH	ISDN131324	ISDN131272	ISDN131353		ISDN125704		ISDN125595	ISDN125650	HK821	HK821	HK821		HK821		HK821	HK821
2005	Ck/VN/NCVDCDC29/05	NCVD		BAC NINH		ISDN131273	ISDN125544	ISDN124081	ISDN125705	ISDN124116	ISDN125596	ISDN125651		GX4016	GX4016	E319(GX401	E319(FJ584)(	GX4016(E319)	E319(GX4016	F1(GX4016)
2005	Ck/VN/NCVDCDC3/05	NCVD	VN3	HA TAY	ISDN131306	ISDN131251	ISDN125524	ISDN124065	ISDN125683	ISDN124100	ISDN125578	ISDN125626	HK821	HK821	HK821	HK821	HK821	HK821	HK821	HK821
2005	Ck/VN/NCVDCDC30/05	NCVD		THAI NGUYEN		ISDN131274	ISDN125545	ISDN124082	ISDN125706	ISDN124117		ISDN125652		HK821	HK821	HK821	HK821	HK821		HK821
2005	Ck/VN/NCVDCDC31/05	NCVD		LAO CAI	ISDN131325	ISDN131275	ISDN125546	ISDN124083	ISDN125707	ISDN124118	ISDN125597		HK821	HK821	HK821	HK821	HK821	HK821	HK821	
2005	Ck/VN/NCVDCDC32/05	NCVD		QUANG BINH	ISDN131326	ISDN131276	ISDN131354	ISDN131239	ISDN125708			ISDN125653	HK821	HK821	HK821	HK821	HK821			HK821
2005	Ck/VN/NCVDCDC33/05	NCVD		NGHE AN		ISDN131277	ISDN125547	ISDN124157	ISDN125709	ISDN124169	ISDN125598	ISDN125654		HK821	HK821	HK821	HK821	HK821	HK821	HK821
2005	Ck/VN/NCVDCDC34/05	NCVD	VN4		ISDN131327	ISDN131278	ISDN125548	ISDN124158	ISDN125710	ISDN124170	ISDN125599	ISDN125655	F1(GX4016)	GX4016	GX4016	E319(GX401	E319(FJ584)(	GX4016(E319)	E319(GX4016	F1(GX4016)
2005	Ck/VN/NCVDCDC36/05	NCVD	VN4	PHU THO	ISDN131328	ISDN131280	ISDN125549	ISDN124084	ISDN125712	ISDN124119	ISDN125601	ISDN125657	F1(GX4016)	GX4016	GX4016	E319(GX401	E319(FJ584)(	GX4016(E319)	E319(GX4016	F1(GX4016)
2005	Ck/VN/NCVDCDC37/05	NCVD	VN3	PHU THO	ISDN131329	ISDN131281	ISDN125550	ISDN124085	ISDN125713	ISDN124120	ISDN125602	ISDN125658	HK821	HK821	HK821	HK821	HK821	HK821	HK821	HK821
2005	Ck/VN/NCVDCDC38/05	NCVD	VN3	THAI BINH	ISDN131330	ISDN131282	ISDN125551	ISDN124086	ISDN125714	ISDN124121	ISDN125603	ISDN125659	HK821	HK821	HK821	HK821	HK821	HK821	HK821	HK821
2005	Ck/VN/NCVDCDC40/05	NCVD	VN3	HAI PHONG	ISDN131332	ISDN131284	ISDN125553	ISDN124088	ISDN125716	ISDN124123	ISDN125605	ISDN125661	HK821	HK821	HK821	HK821	HK821	HK821	HK821	HK821
2005	Ck/VN/NCVDCDC42/05	NCVD	VN3	HAI PHONG	ISDN131334	ISDN131286	ISDN125555	ISDN124090	ISDN125718	ISDN124125	ISDN125607	ISDN125663	HK821	HK821	HK821	HK821	HK821	HK821	HK821	HK821
2005	Ck/VN/NCVDCDC43/05	NCVD	VN3	HAI PHONG	ISDN131335	ISDN131287	ISDN125556	ISDN124091	ISDN125719	ISDN124126	ISDN125608	ISDN125664	HK821	HK821	HK821	HK821	HK821	HK821	HK821	HK821
2005	Ck/VN/NCVDCDC44/05	NCVD		HAI PHONG		ISDN131288	ISDN125557	ISDN124092	ISDN125720	ISDN124127	ISDN125609	ISDN125665		HK821	HK821	HK821	HK821	HK821	HK821	HK821
2005	Ck/VN/NCVDCDC45/05	NCVD		HA GIANG		ISDN131289	ISDN125558	ISDN124093	ISDN125721	ISDN124128	ISDN125610	ISDN125666		HK821	HK821	HK821	HK821	HK821	HK821	HK821
2005	Ck/VN/NCVDCDC49/05	NCVD		YEN BAI	ISDN131336	ISDN131293	ISDN131356	ISDN131240	ISDN125725		ISDN125614	ISDN125670	HK821	HK821	HK821	HK821	HK821		HK821	HK821
2005	Ck/VN/NCVDCDC51/05	NCVD		HA NOI		ISDN131295	ISDN125562	ISDN124159	ISDN125727	ISDN124171	ISDN125616	ISDN125672		HK821	HK821	HK821	HK821	HK821	HK821	HK821
2005	Ck/VN/NCVDCDC52/05	NCVD	VN3	VINH PHUC	ISDN131337	ISDN131296	ISDN125563	ISDN124160	ISDN125728	ISDN124172	ISDN125617	ISDN125673	HK821	HK821	HK821	HK821	HK821	HK821	HK821	HK821
2005	Dk/VN/NCVD01/05	NCVD						EF566203		EF566208						HK821		HK821		
2005	Dk/VN/NCVD03/05	NCVD								EF566210								HK821		

2005	Dk/VN/NCVD04/05	NCVD						EF566201		EF566211						HK821		HK821		
2005	Dk/VN/NCVD05/05	NCVD						EF566204		EF566205						HK821		HK821		
2005	Dk/VN/NCVD06/05	NCVD						EF566197								HK821				
2005	Dk/VN/NCVD07/05	NCVD		VINH PHUC				EF566196		EF566206						HK821		HK821		
2005	Dk/VN/NCVD08/05	NCVD		VINH PHUC				EF566195		EF566207						HK821		HK821		
2005	Dk/VN/NCVDCDC10/05	NCVD	VN3	AN GIANG	ISDN131310	ISDN131256	ISDN125529	ISDN124069	ISDN125689	ISDN124104	ISDN125582	ISDN125633	HK821	HK821	HK821	HK821	HK821	HK821	HK821	HK821
2005	Dk/VN/NCVDCDC11/05	NCVD	VN3	AN GIANG	ISDN131311	ISDN131257	ISDN125530	ISDN124070	ISDN125690	ISDN124105	ISDN125583	ISDN125634	HK821	HK821	HK821	HK821	HK821	HK821	HK821	HK821
2005	Dk/VN/NCVDCDC12/05	NCVD	VN3	AN GIANG	ISDN131312	ISDN131258	ISDN125531	ISDN124071	ISDN125691	ISDN124106	ISDN125584	ISDN125635	HK821	HK821	HK821	HK821	HK821	HK821	HK821	HK821
2005	Dk/VN/NCVDCDC13/05	NCVD	VN4	TIEN GIANG	ISDN131313	ISDN131259	ISDN125532	ISDN124072	ISDN125692	ISDN124107	ISDN125585	ISDN125636	F1(GX4016)	GX4016	GX4016	E319(GX401	E319(FJ584)(	GX4016(E319)	E319(GX4016	F1(GX4016)
2005	Dk/VN/NCVDCDC14/05	NCVD	VN3	TIEN GIANG	ISDN131314	ISDN131260	ISDN125533	ISDN124073	ISDN125693	ISDN124108	ISDN125586	ISDN125637	HK821	HK821	HK821	HK821	HK821	HK821	HK821	HK821
2005	Dk/VN/NCVDCDC15/05	NCVD		TIEN GIANG		ISDN131261	ISDN125534	ISDN124074	ISDN125694	ISDN124109	ISDN125587	ISDN125638		HK821	HK821	HK821	HK821	HK821	HK821	HK821
2005	Dk/VN/NCVDCDC16/05	NCVD	VN4	TIEN GIANG	ISDN131315	ISDN131262	ISDN125535	ISDN124075	ISDN125695	ISDN124110	ISDN125588	ISDN125639	F1(GX4016)	GX4016	GX4016	E319(GX401	E319(FJ584)(	GX4016(E319)	E319(GX4016	F1(GX4016)
2005	Dk/VN/NCVDCDC17/05	NCVD		HA NOI		ISDN131263	ISDN125536	ISDN124154	ISDN125696	ISDN124166		ISDN125640		HK821	HK821	HK821	HK821	HK821		HK821
2005	Dk/VN/NCVDCDC2/05	NCVD	VN3	HA TAY	ISDN131305	ISDN131250	ISDN125523	ISDN124064	ISDN125682	ISDN124099	ISDN125577	ISDN125625	HK821	HK821	HK821	HK821	HK821	HK821	HK821	HK821
2005	Dk/VN/NCVDCDC39/05	NCVD	VN3	THAI BINH	ISDN131331	ISDN131283	ISDN125552	ISDN124087	ISDN125715	ISDN124122	ISDN125604	ISDN125660	HK821	HK821	HK821	HK821	HK821	HK821	HK821	HK821
2005	Dk/VN/NCVDCDC4/05	NCVD		HA TAY		ISDN131252	ISDN125525	ISDN124066	ISDN125684	ISDN124101	ISDN125579	ISDN125627		HK821	HK821	HK821	HK821	HK821	HK821	HK821
2005	Dk/VN/NCVDCDC41/05	NCVD	VN3	HAI PHONG	ISDN131333	ISDN131285	ISDN125554	ISDN124089	ISDN125717	ISDN124124	ISDN125606	ISDN125662	HK821	HK821	HK821	HK821	HK821	HK821	HK821	HK821
2005	Dk/VN/NCVDCDC46/05	NCVD		NAM DINH		ISDN131290	ISDN125559	ISDN124094	ISDN125722	ISDN124129	ISDN125611	ISDN125667		GX4016	GX4016	E319(GX401	E319(FJ584)(	GX4016(E319)	E319(GX4016	F1(GX4016)
2005	Dk/VN/NCVDCDC47/05	NCVD		NAM DINH		ISDN131291	ISDN125560	ISDN124095	ISDN125723	ISDN124130	ISDN125612	ISDN125668		HK821	HK821	HK821	HK821	HK821	HK821	HK821
2005	Dk/VN/NCVDCDC48/05	NCVD		THANH HOA		ISDN131292	ISDN125561	ISDN124096	ISDN125724	ISDN124131	ISDN125613	ISDN125669		HK821	HK821	HK821	HK821	HK821	HK821	HK821
2005	Dk/VN/NCVDCDC5/05	NCVD		VINH PHUC	ISDN131307	ISDN131253	ISDN131349		ISDN125685		ISDN125580	ISDN125628	HK821	HK821	HK821		HK821		HK821	HK821
2005	Dk/VN/NCVDCDC50/05	NCVD		QUANG NINH		ISDN131294	ISDN131357		ISDN125726		ISDN125615	ISDN125671		GX4016	GX4016		E319(FJ584)(GX4016)		E319(GX4016	F1(GX4016)
2005	Dk/VN/NCVDCDC6/05	NCVD		VINH PHUC			ISDN125526	ISDN124067	ISDN125686	ISDN124102		ISDN125629			HK821	HK821	HK821	HK821		HK821
2005	Dk/VN/NCVDCDC61/05	NCVD	VN3		ISDN127514	ISDN127526	ISDN127539	ISDN127552	ISDN127564	ISDN127577	ISDN127590	ISDN127603	HK821	HK821	HK821	HK821	HK821	HK821	HK821	HK821
2005	Dk/VN/NCVDCDC62/05	NCVD	VN3		ISDN131345	ISDN127527	ISDN127540	ISDN127553	ISDN127565	ISDN127578	ISDN127591	ISDN127604	HK821	HK821	HK821	HK821	HK821	HK821	HK821	HK821
2005	Dk/VN/NCVDCDC63/05	NCVD	VN3	AN GIANG	ISDN131346	ISDN127528	ISDN127541	ISDN127554	ISDN127566	ISDN127579	ISDN127592	ISDN127605	HK821	HK821	HK821	HK821	HK821	HK821	HK821	HK821
2005	Dk/VN/NCVDCDC64/05	NCVD	VN3	NINH BINH	ISDN127517	ISDN127529	ISDN127542	ISDN127555	ISDN127567	ISDN127580	ISDN127593	ISDN127606	HK821	HK821	HK821	HK821	HK821	HK821	HK821	HK821
2005	Dk/VN/NCVDCDC65/05	NCVD				ISDN127530	ISDN127543	ISDN127556	ISDN127568	ISDN127581	ISDN127594	ISDN127607		HK821	HK821	HK821	HK821	HK821	HK821	HK821
2005	Dk/VN/NCVDCDC69/05	NCVD	VN3		ISDN127518	ISDN127531	ISDN127544	ISDN127557	ISDN127569	ISDN127582	ISDN127595	ISDN127608	HK821	HK821	HK821	HK821	HK821	HK821	HK821	HK821
2005	Dk/VN/NCVDCDC7/05	NCVD					ISDN131350					ISDN125630			HK821					HK821
2005	Dk/VN/NCVDCDC75/05	NCVD	VN4		ISDN127519	ISDN127532	ISDN127545	ISDN127558	ISDN127570	ISDN127583	ISDN127596	ISDN127609	F1(GX4016)	GX4016	GX4016	E319(GX401	E319(FJ584)(	GX4016(E319)	E319(GX4016	F1(GX4016)
2005	Dk/VN/NCVDCDC78/05	NCVD	VN4		ISDN127520	ISDN127533	ISDN127546	ISDN127559	ISDN127571	ISDN127584	ISDN127597	ISDN127610	F1(GX4016)	GX4016	GX4016	E319(GX401	E319(FJ584)(	GX4016(E319)	E319(GX4016	F1(GX4016)
2005	Dk/VN/NCVDCDC79/05	NCVD			ISDN127521	ISDN127534	ISDN127547		ISDN127572	ISDN127585	ISDN127598	ISDN127611	F1(GX4016)	GX4016	GX4016		E319(FJ584)(	GX4016(E319)	E319(GX4016	F1(GX4016)
2005	Dk/VN/NCVDCDC8/05	NCVD			ISDN131308	ISDN131254	ISDN125527	ISDN124153	ISDN125687	ISDN124165		ISDN125631	HK821	HK821	HK821	HK821	HK821	HK821		HK821
2005	Dk/VN/NCVDCDC9/05	NCVD	VN3		ISDN131309	ISDN131255	ISDN125528	ISDN124068	ISDN125688	ISDN124103	ISDN125581	ISDN125632	HK821	HK821	HK821	HK821	HK821	HK821	HK821	HK821
2005	Dk/VN/NCVDCDC93/05	NCVD	VN4		ISDN127523	ISDN127536	ISDN127549	ISDN127561	ISDN127574	ISDN127587	ISDN127600	ISDN127613	F1(GX4016)	GX4016	GX4016	E319(GX401	E319(FJ584)(	GX4016(E319)	E319(GX4016	F1(GX4016)
2005	Dk/VN/NCVDCDC94/05	NCVD	VN4		ISDN127524	ISDN127537	ISDN127550	ISDN127562	ISDN127575	ISDN127588	ISDN127601	ISDN127614	F1(GX4016)	GX4016	GX4016	E319(GX401	E319(FJ584)(	GX4016(E319)	E319(GX4016	F1(GX4016)
2005	Dk/VN/NCVDCDC95/05	NCVD	VN4		ISDN127525	ISDN127538	ISDN127551	ISDN127563	ISDN127576	ISDN127589	ISDN127602	ISDN127615	F1(GX4016)	GX4016	GX4016	E319(GX401	E319(FJ584)(	GX4016(E319)	E319(GX4016	F1(GX4016)
2005	ENV/VN/NCVD109/05	NCVD					ISDN131360	ISDN131242							GX4016	E319(GX401				
2005	ENV/VN/NCVDCDC53/05	NCVD		HA TAY	ISDN131338	ISDN131297	ISDN131358		ISDN125729		ISDN125618	ISDN125674	HK821	HK821	HK821		HK821		HK821	HK821
2005	ENV/VN/NCVDCDC54/05	NCVD	VN3	HA TAY	ISDN131339	ISDN131298	ISDN125564	ISDN124161	ISDN125730	ISDN124173	ISDN125619	ISDN125675	HK821	HK821	HK821	HK821	HK821	HK821	HK821	HK821

2005	ENV/VN/NCVDCDC55/05	NCVD	VN3	VINH PHUC	ISDN131340	ISDN131299	ISDN125565	ISDN124097	ISDN125731	ISDN124132	ISDN125680	ISDN125676	HK821	HK821	HK821	HK821	HK821	HK821	HK821	HK821
2005	ENV/VN/NCVDCDC56/05	NCVD	VN3	TIEN GIANG	ISDN131341	ISDN131300	ISDN125566	ISDN124162	ISDN125732	ISDN124174	ISDN125620	ISDN125677	HK821	HK821	HK821	HK821	HK821	HK821	HK821	HK821
2005	ENV/VN/NCVDCDC59/05	NCVD		TIEN GIANG	ISDN131342	ISDN131301	ISDN131359	ISDN124163		ISDN124175	ISDN125621	ISDN125678	HK821	HK821	HK821	HK821		HK821	HK821	HK821
2005	ENV/VN/NCVDCDC60/05	NCVD			ISDN131343	ISDN131302	ISDN125567	ISDN124164		ISDN124176	ISDN125622	ISDN125679	HK821	HK821	HK821	HK821		HK821	HK821	HK821
2005	M.Dk/VN/NCVD02/05	NCVD						EF566202		EF566209						HK821		HK821		
2005	M.Dk/VN/NCVDCDC35/05	NCVD		NINH BINH		ISDN131279	ISDN131355		ISDN125711		ISDN125600	ISDN125656		HK821	HK821		HK821		HK821	HK821
2005	M.Dk/VN/NCVDCDC89/05	NCVD	VN3		ISDN127522	ISDN127535	ISDN127548	ISDN127560	ISDN127573	ISDN127586	ISDN127599	ISDN127612	HK821	HK821	HK821	HK821	HK821	HK821	HK821	HK821
2005	PEACOCK/VN/NCVD110/05	NCVD		NINH BINH		ISDN131303		ISDN131243						GX4016		E319(GX4016)(GX604)				
2005	QUAIL/VN/NCVD118/05	NCVD		HA NOI				ISDN131245								E319(GX4016)(GX604)				
2005	SPARROW/VN/NCVD137/05	NCVD		VINH PHUC	ISDN131348			ISDN131246					F1(GX4016)			E319(GX4016)(GX604)				
2007	Ck/LAOS/NCVD-38/07*	NCVD	VN6	LAO PDR		CY030483		CY030485	CY030486	CY030487	CY030488	CY030489		FJ584(E31		FJ584	E319(FJ584)((	FJ584	FJ584	FJ584
2007	Ck/VN/NCVD-10/07	NCVD	VN3	BAC LIEU	CY030308	CY030309	CY030310	CY030311	CY030312	CY030313	CY030314	CY030315	HK821	HK821	HK821	HK821	HK821	HK821	HK821	HK821
2007	Ck/VN/NCVD-15/07	NCVD	VN3	HAU GIANG	CY030340	CY030341	CY030342	CY030343	CY030344	CY030345	CY030346	CY030347	HK821	HK821	HK821	HK821	HK821	HK821	HK821	HK821
2007	Ck/VN/NCVD-20/07	NCVD	VN7	HA TAY	CY030380	CY030381	CY030382	CY030383	CY030384	CY030385	CY030386	CY030387	GX604(E319)	FJ584(E31	FJ584	FJ584	E319(FJ584)((	GX4016(E319)	FJ584	FJ584
2007	Ck/VN/NCVD-21/07	NCVD	VN3	HA TAY	CY030388	CY030389	CY030390	CY030391	CY030392	CY030393	CY030394	CY030395	HK821	HK821	HK821	HK821	HK821	HK821	HK821	HK821
2007	Ck/VN/NCVD-24/07	NCVD	VN3	HAU GIANG	CY030412	CY030413	CY030414	CY030415	CY030416	CY030417	CY030418	CY030419	HK821	HK821	HK821	HK821	HK821	HK821	HK821	HK821
2007	Ck/VN/NCVD-3/07	NCVD	VN3	CA MAU	CY030252	CY030253	CY030254	CY030255	CY030256	CY030257	CY030258	CY030259	HK821	HK821	HK821	HK821	HK821	HK821	HK821	HK821
2007	Ck/VN/NCVD-40/07	NCVD	VN7	HAI DUONG	CY030498	CY030499	CY030500	CY030501	CY030502	CY030503	CY030504	CY030505	GX604(E319)	FJ584(E31	FJ584	FJ584	E319(FJ584)((	GX4016(E319)	FJ584	FJ584
2007	Ck/VN/NCVD-41/07	NCVD		HAI DUONG	CY030506	CY030507		CY030509	CY030510	CY030511	CY030512	CY030513	GX604(E319)	FJ584(E31		FJ584	E319(FJ584)((	GX4016(E319)	FJ584	FJ584
2007	Ck/VN/NCVD-42/07	NCVD		HA NOI	CY030723	CY030514		CY030515	CY030516	CY030517	CY030518	CY030519	GX604(E319)	FJ584(E31		FJ584	E319(FJ584)((	GX4016(E319)	FJ584	FJ584
2007	Ck/VN/NCVD-44/07	NCVD	VN6	HA TAY	CY030528	CY030529	CY030530	CY030531	CY030532	CY030533	CY030534	CY030535	GX604(E319)	FJ584(E31	FJ584	FJ584	E319(FJ584)((	FJ584	FJ584	FJ584
2007	Ck/VN/NCVD-45/07	NCVD	VN6	HA NOI	CY030536	CY030537	CY030724	CY030538	CY030539	CY030540	CY030541	CY030542	GX604(E319)	FJ584(E31	FJ584	FJ584	E319(FJ584)((	FJ584	FJ584	FJ584
2007	Dk/LAOS/NCVD-35/07*	NCVD	VN6	LAO PDR	CY030466	CY030467	CY030468	CY030469	CY030470	CY030471	CY030472	CY030473	GX604(E319)	FJ584(E31	FJ584	FJ584	E319(FJ584)((	FJ584	FJ584	FJ584
2007	Dk/VN/NCVD-1/07	NCVD	VN3	CA MAU	CY030236	CY030237	CY030238	CY030239	CY030240	CY030241	CY030242	CY030243	HK821	HK821	HK821	HK821	HK821	HK821	HK821	HK821
2007	Dk/VN/NCVD-12/07	NCVD	VN3	HAU GIANG	CY030324	CY030325	CY030326	CY030327	CY030328	CY030329	CY030330	CY030331	HK821	HK821	HK821	HK821	HK821	HK821	HK821	HK821
2007	Dk/VN/NCVD-13/07	NCVD	VN3	HAU GIANG	CY030332	CY030333	CY030334	CY030335	CY030336	CY030337	CY030338	CY030339	HK821	HK821	HK821	HK821	HK821	HK821	HK821	HK821
2007	Dk/VN/NCVD-16/07	NCVD	VN3	KIEN GIANG	CY030348	CY030349	CY030350	CY030351	CY030352	CY030353	CY030354	CY030355	HK821	HK821	HK821	HK821	HK821	HK821	HK821	HK821
2007	Dk/VN/NCVD-17/07	NCVD	VN3	KIEN GIANG	CY030356	CY030357	CY030358	CY030359	CY030360	CY030361	CY030362	CY030363	HK821	HK821	HK821	HK821	HK821	HK821	HK821	HK821
2007	Dk/VN/NCVD-18/07	NCVD	VN3	KIEN GIANG	CY030364	CY030365	CY030366	CY030367	CY030368	CY030369	CY030370	CY030371	HK821	HK821	HK821	HK821	HK821	HK821	HK821	HK821
2007	Dk/VN/NCVD-19/07	NCVD	VN3	KIEN GIANG	CY030372	CY030373	CY030374	CY030375	CY030376	CY030377	CY030378	CY030379	HK821	HK821	HK821	HK821	HK821	HK821	HK821	HK821
2007	Dk/VN/NCVD-2/07	NCVD	VN3	CA MAU	CY030244	CY030245	CY030246	CY030247	CY030248	CY030249	CY030250	CY030251	HK821	HK821	HK821	HK821	HK821	HK821	HK821	HK821
2007	Dk/VN/NCVD-25/07	NCVD	VN3	SOC TRANG	CY030420	CY030421	CY030422	CY030423	CY030424	CY030425	CY030426	CY030427	HK821	HK821	HK821	HK821	HK821	HK821	HK821	HK821
2007	Dk/VN/NCVD-26/07	NCVD	VN3	VINH LONG	CY030428	CY030429	CY030430	CY030431	CY030432	CY030433	CY030434	CY030435	HK821	HK821	HK821	HK821	HK821	HK821	HK821	HK821
2007	Dk/VN/NCVD-30/07	NCVD	VN3	KIEN GIANG	CY030443	CY030444	CY030722	CY030445	CY030446	CY030447	CY030448	CY030449	HK821	HK821	HK821	HK821	HK821	HK821	HK821	HK821
2007	Dk/VN/NCVD-31/07	NCVD	VN6	SOC TRANG	CY030450	CY030451	CY030452	CY030453	CY030454	CY030455	CY030456	CY030457	GX604(E319)	FJ584(E31	FJ584	FJ584	E319(FJ584)((	FJ584	FJ584	FJ584
2007	Dk/VN/NCVD-34/07	NCVD		HA TAY	CY030458	CY030459		CY030461	CY030462	CY030463	CY030464	CY030465	GX604(E319)	FJ584(E31		FJ584	E319(FJ584)((	GX4016(E319)	FJ584	FJ584
2007	Dk/VN/NCVD-43/07	NCVD	VN9	HA NOI	CY030520	CY030521	CY030522	CY030523	CY030524	CY030525	CY030526	CY030527	HK821	FJ584(E31	HK821	FJ584	HK821	HK821	HK821	HK821
2007	Dk/VN/NCVD-6/07	NCVD	VN3	BAC LIEU	CY030276	CY030277	CY030278	CY030279	CY030280	CY030281	CY030282	CY030283	HK821	HK821	HK821	HK821	HK821	HK821	HK821	HK821
2007	Dk/VN/NCVD-7/07	NCVD	VN3	BAC LIEU	CY030284	CY030285	CY030286	CY030287	CY030288	CY030289	CY030290	CY030291	HK821	HK821	HK821	HK821	HK821	HK821	HK821	HK821
2007	Dk/VN/NCVD-8/07	NCVD	VN3	BAC LIEU	CY030292	CY030293	CY030294	CY030295	CY030296	CY030297	CY030298	CY030299	HK821	HK821	HK821	HK821	HK821	HK821	HK821	HK821
2007	Dk/VN/NCVD-9/07	NCVD	VN3	BAC LIEU	CY030300	CY030301	CY030302	CY030303	CY030304	CY030305	CY030306	CY030307	HK821	HK821	HK821	HK821	HK821	HK821	HK821	HK821

2007	Gs/LAOS/NCVD-39/07*	NCVD	VN6	LAO PDR	CY030490	CY030491	CY030492	CY030493	CY030494	CY030495	CY030496	CY030497	GX604(E319)	FJ584(E31	FJ584(E31	FJ584	E319(FJ584)(	FJ584	FJ584	FJ584
2007	M.Dk/VN/NCVD-11/07	NCVD	VN3	BAC LIEU	CY030316	CY030317	CY030318	CY030319	CY030320	CY030321	CY030322	CY030323	HK821	HK821	HK821	HK821	HK821	HK821	HK821	HK821
2007	M.Dk/VN/NCVD-22/07	NCVD	VN8	CA MAU	CY030396	CY030397	CY030398	CY030399	CY030400	CY030401	CY030402	CY030403	GX604(E319)	FJ584(E31	HK821	FJ584	HK821	GX4016(E319)	FJ584	HK821
2007	M.Dk/VN/NCVD-23/07	NCVD	VN3	CA MAU	CY030404	CY030405	CY030406	CY030407	CY030408	CY030409	CY030410	CY030411	HK821	HK821	HK821	HK821	HK821	HK821	HK821	HK821
2007	M.Dk/VN/NCVD-29/07	NCVD	VN3	CA MAU	CY030436	CY030437	CY030721	CY030438	CY030439	CY030440	CY030441	CY030442	HK821	HK821	HK821	HK821	HK821	HK821	HK821	HK821
2007	M.Dk/VN/NCVD-4/07	NCVD	VN3	CA MAU	CY030260	CY030261	CY030262	CY030263	CY030264	CY030265	CY030266	CY030267	HK821	HK821	HK821	HK821	HK821	HK821	HK821	HK821
2007	M.Dk/VN/NCVD-46/07	NCVD	VN6	HAI DUONG	CY030725	CY030543	CY030726	CY030544	CY030545	CY030546	CY030547	CY030548	GX604(E319)	FJ584	FJ584	FJ584	E319(FJ584)(	FJ584	FJ584	FJ584
2007	M.Dk/VN/NCVD-5/07	NCVD	VN3	CA MAU	CY030268	CY030269	CY030270	CY030271	CY030272	CY030273	CY030274	CY030275	HK821	HK821	HK821	HK821	HK821	HK821	HK821	HK821
2007	PIGEON/LAOS/NCVD-36/07*	NCVD		LAO PDR		CY030475		CY030477	CY030478	CY030479	CY030480	CY030481		FJ584(E31		FJ584	E319(FJ584)(	FJ584	FJ584	FJ584

*: Four strains from LAOS.
