# Supplementary material for: Evolution of Highly Pathogenic H5N1 Avian Influenza Viruses in Vietnam between 2001 and 2007
Source: PLoS One. 2008 Oct 21;3(10):e3462. doi: 10.1371/journal.pone.0003462 (PMC2565130; doi:10.1371/journal.pone.0003462)
Supplement: Table S2 — Number of H5N1 isolates collected and percentage of each identified genotype in Vietnam per year. (0.01 MB PDF) [file pone.0003462.s002.pdf]

Table S2. Number of H5N1 isolates collected and percentage of each identified genotype in

Genotype	Year					
	2001	2003	2004	2005	2006	2007
VN1	2 (100%)*					
VN2		2 (4.8%)				
VN3		19 (46.3%)	52 (65.8%)	62 (36.5%)		25 (62.5%)
VN4				15 (8.8%)		
VN5				3 (1.8%)		
VN6						7 (17.5%)
VN7						2 (5%)
VN8						1 (2.5%)
VN9						1 (2.5%)
Unknown***		20 (48.8%)	27 (34.2%)	90 (52.9%)		4 (10%)
Total	2	41	79	170		40

*The number in the parenthesis is the percentage of isolates from each genotype per year.

**The number in the parenthesis is the total percentage of isolates from each genotype from 2001 to 2007.

***Unknown denotes the viral genotype was not defined because complete genome sequences were not available.

Vietnam per year.

Total
2 (0.6%)**
2 (0.6%)
158 (47.6%)
15 (4.5%)
3 (0.9%)
7 (2.1%)
2 (0.6%)
1 (0.3%)
1 (0.3%)
141 (42.5%)
332
