# Supplementary material for: Evolution of Highly Pathogenic H5N1 Avian Influenza Viruses in Vietnam between 2001 and 2007
Source: PLoS One. 2008 Oct 21;3(10):e3462. doi: 10.1371/journal.pone.0003462 (PMC2565130; doi:10.1371/journal.pone.0003462)
Supplement: Table S3 — Geographic origin of HPAI H5N1 viruses from Vietnam. (0.01 MB PDF) [file pone.0003462.s003.pdf]

Table S3. Geographic origin of HPAI H5N1 viruses from Vietnam.

northern Vietnam						southern Vietnam					
Province	2003	2004	2005	2006	2007	Province	2003	2004	2005	2006	2007
Bac Can						An Giang		1(1)	7(3)		
Bac Giang						Ba Ria-Vung Tau					
Bac Ninh	2		4			Bac Lieu		2(2)	2(2)		6
Cao Bang						Ben Tre					
Dien Bien						Binh Dinh		2(2)			
Ha Giang		1(1)*	2(1)			Binh Duong			1		
Ha Nam						Binh Phuoc					
Ha Tay	15(7)	8(7)	7		4	Binh Thuan					
Ha Tinh						Ca Mau		2(2)	1(1)		8
Hai Duong			3		3	Can Tho		4(4)	2(2)		
Haiphong			5			Da Nang					
Hanoi	8(3)	4(4)	15(7)		3	Dac Lac					
Hoa Binh						Dac Nong					
Hung Yen	3					Dong Nai		2(2)			
Lai Chau						Dong Thap		3(3)	9(9)		
Lang Son						Gia Lai					
Lao Cai			1			Hau Giang		4(4)	4(4)		5
Nam Dinh			3			Ho Chi Minh		2(2)			
Nghe An			1			Khanh Hoa		1(1)			
Ninh Binh			4			Kien Giang		1(1)	2(2)		5
Phu Tho	1		2			Kon Tum					
Quang Binh	1		1			Lam Dong		4(4)			
Quang Ninh			1			Long An		1(1)	2(1)		
Son La						Ninh Thuan					
Thai Binh		1(1)	4(2)			Phu Yen					
Thai Nguyen			4(3)			Quang Nam					
Thanh Hoa			1			Quang Ngai					
Tuyen Quang			2(2)			Quang Tri					
Vinh Phuc	10(4)		7			Soc Trang		2(2)	2		2
Yen Bai			1			Tay Ninh		2(2)			
						Thua Thien-Hue					
						Tien Giang		4(4)	6		
						Tra Vinh		3(3)	2(2)		
						Vinh Long		2(2)	2(2)		1

*The numbers in parentheses indicate those viruses were from public databases.
