# Supplementary material for: Evolution of Highly Pathogenic H5N1 Avian Influenza Viruses in Vietnam between 2001 and 2007
Source: PLoS One. 2008 Oct 21;3(10):e3462. doi: 10.1371/journal.pone.0003462 (PMC2565130; doi:10.1371/journal.pone.0003462)

Figure S1A HA

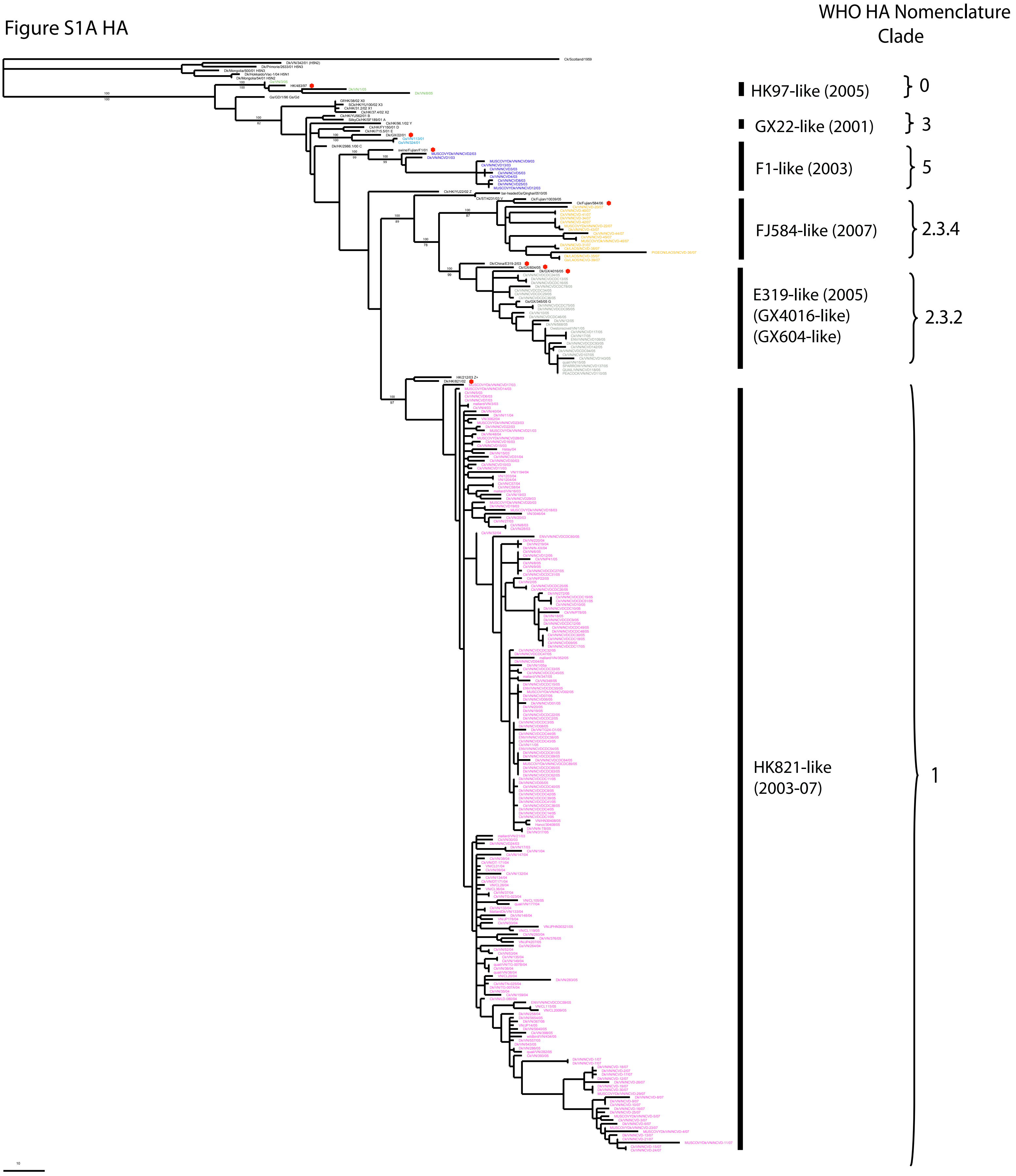

Figure S1B NA

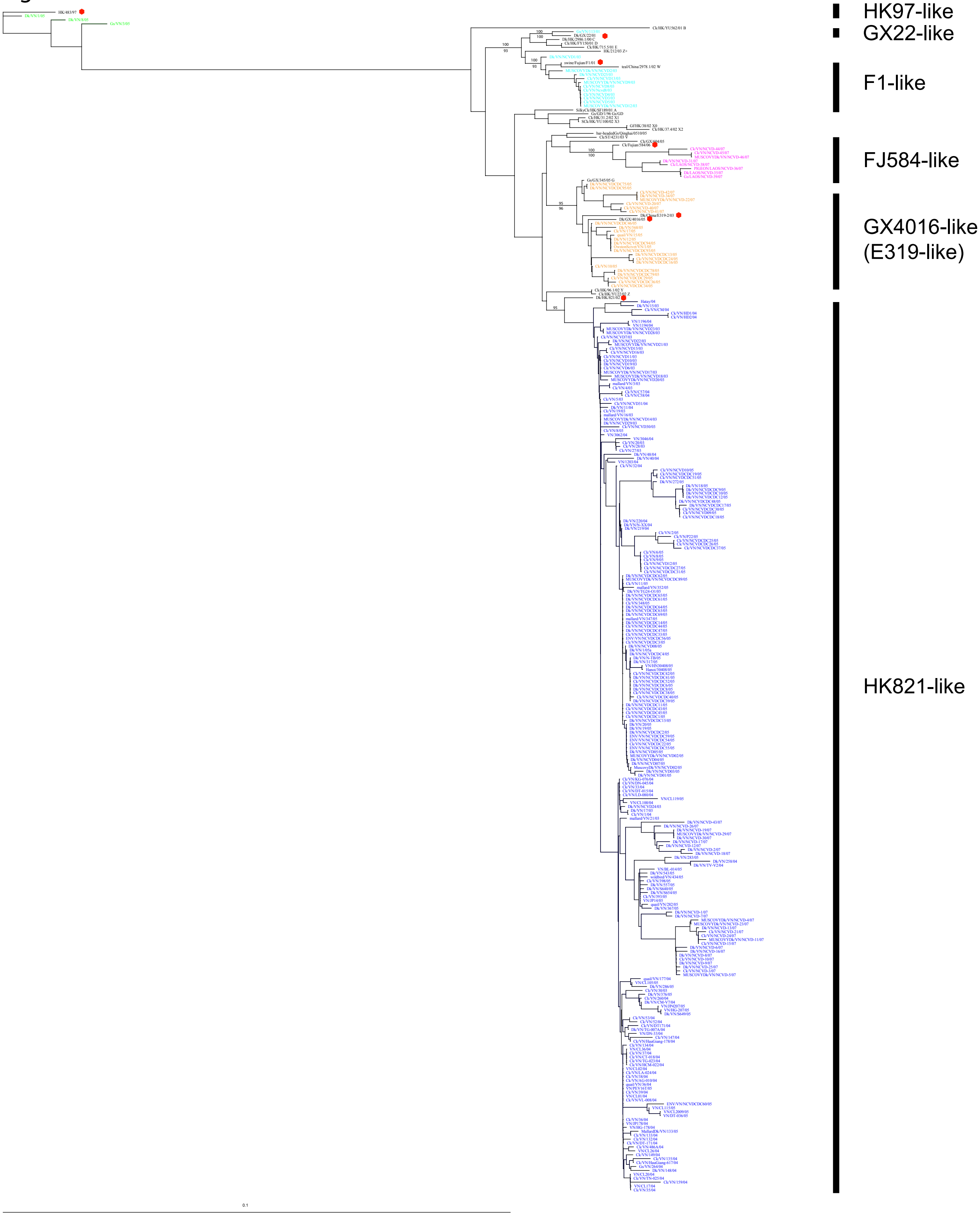

Figure S1C PB2

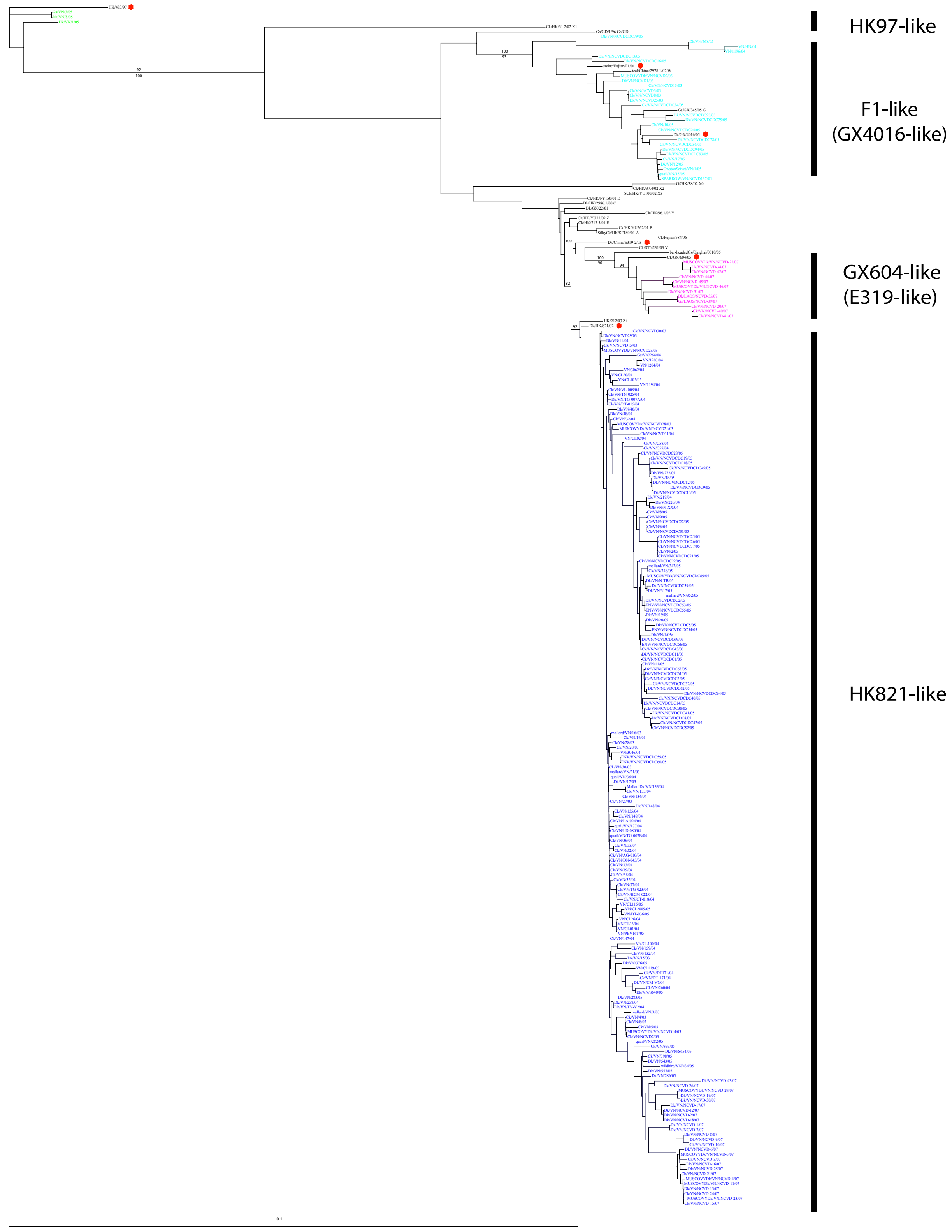

Figure S1D PB1

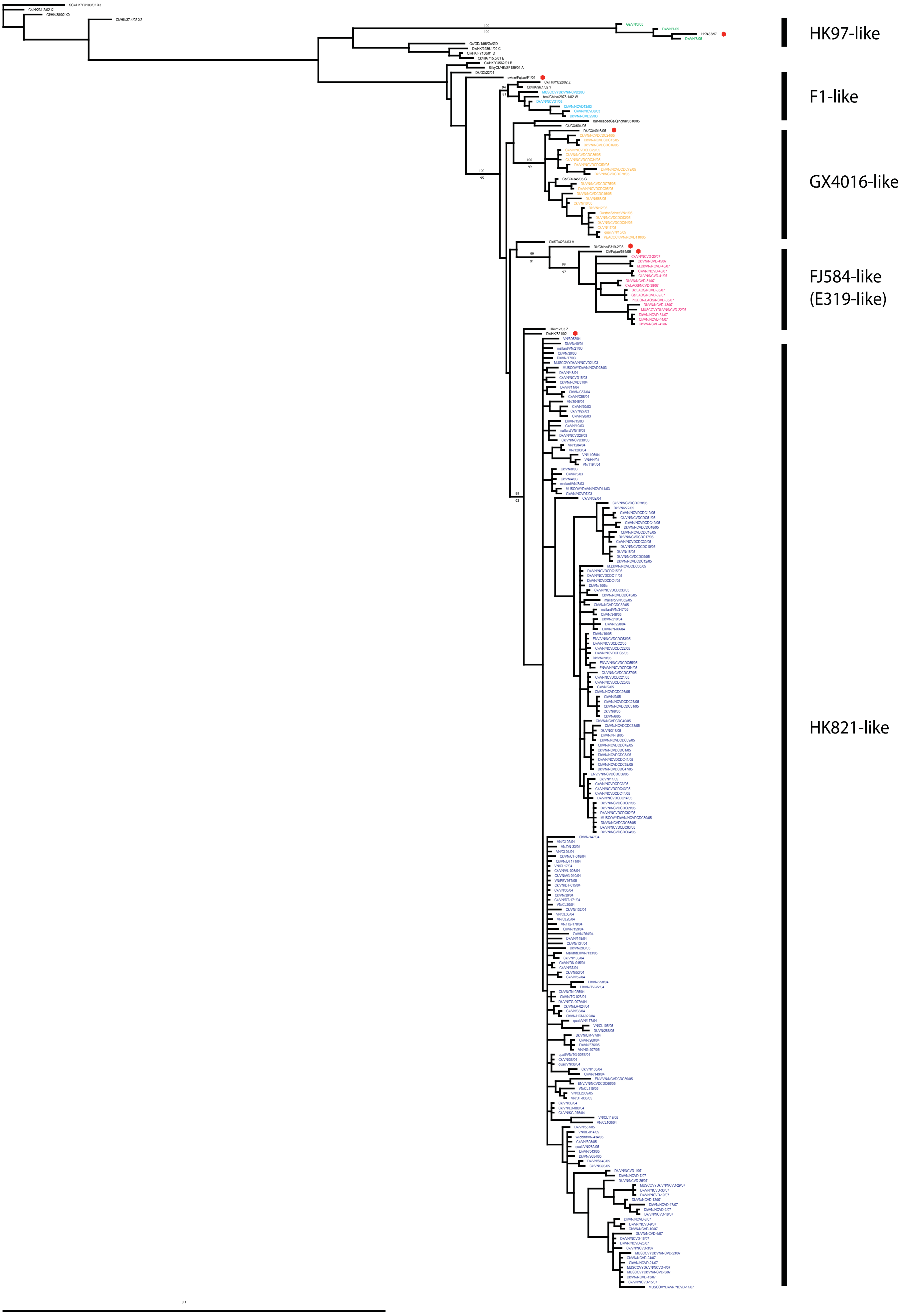

Figure S1E PA

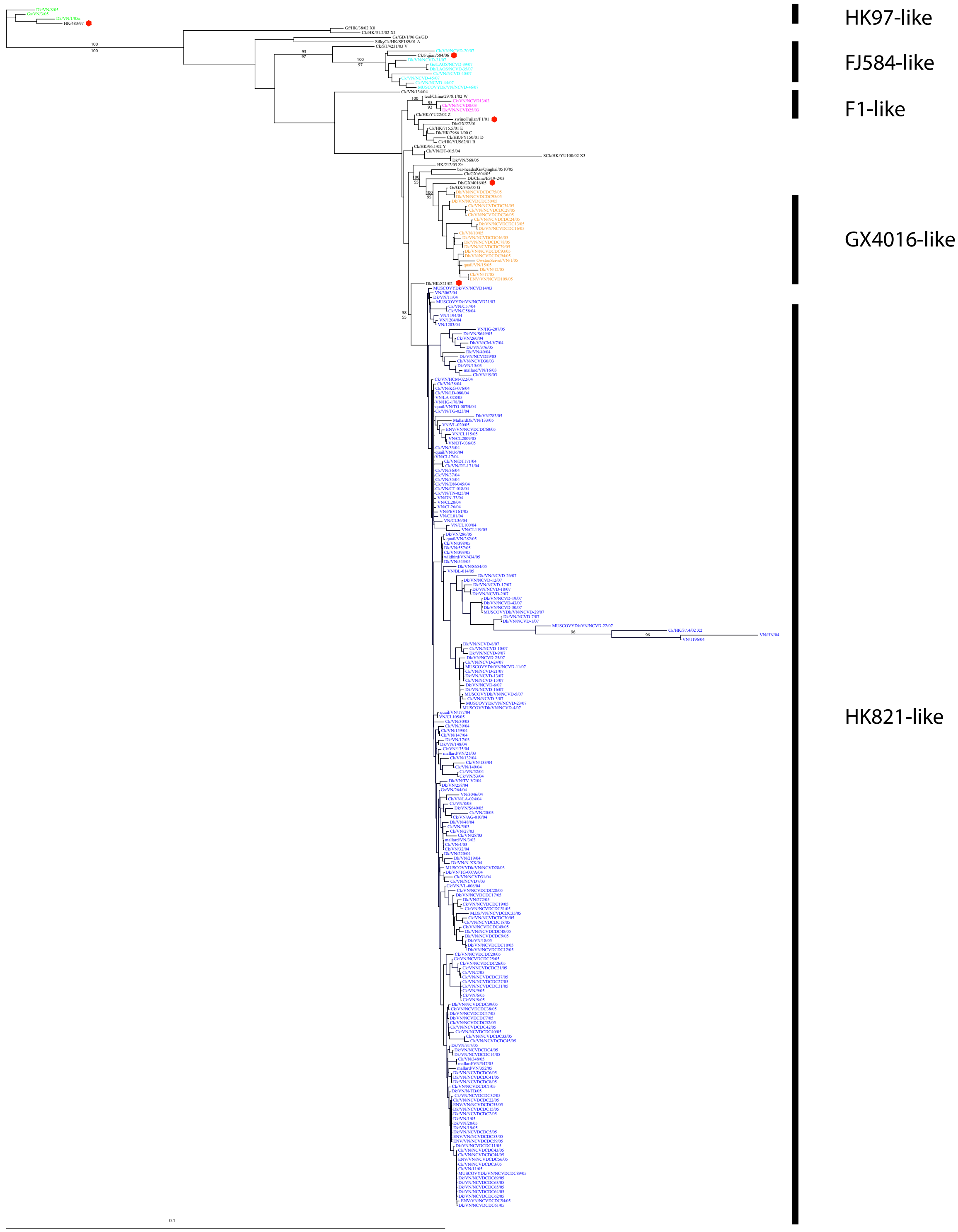

Figure S1F NP

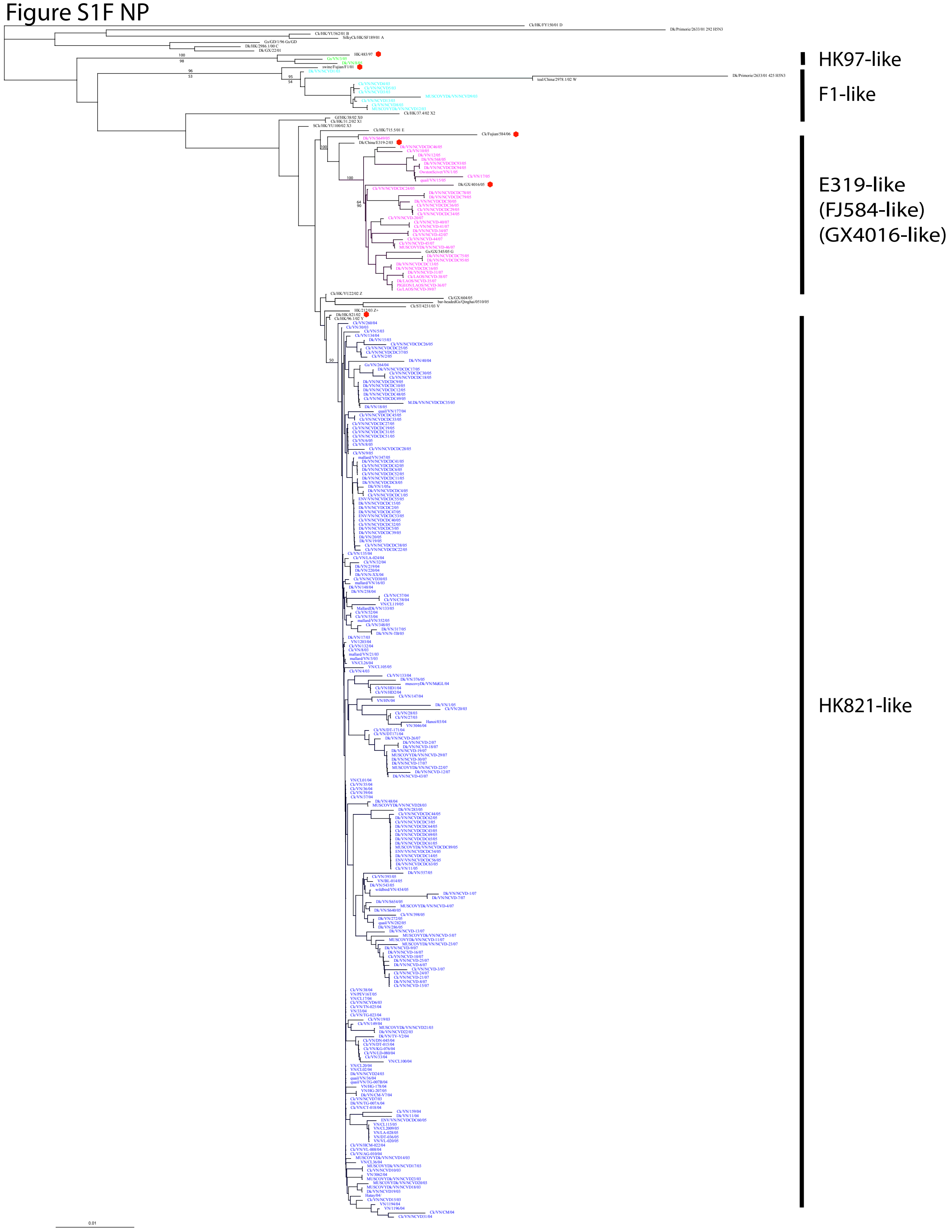

Figure S1G MP

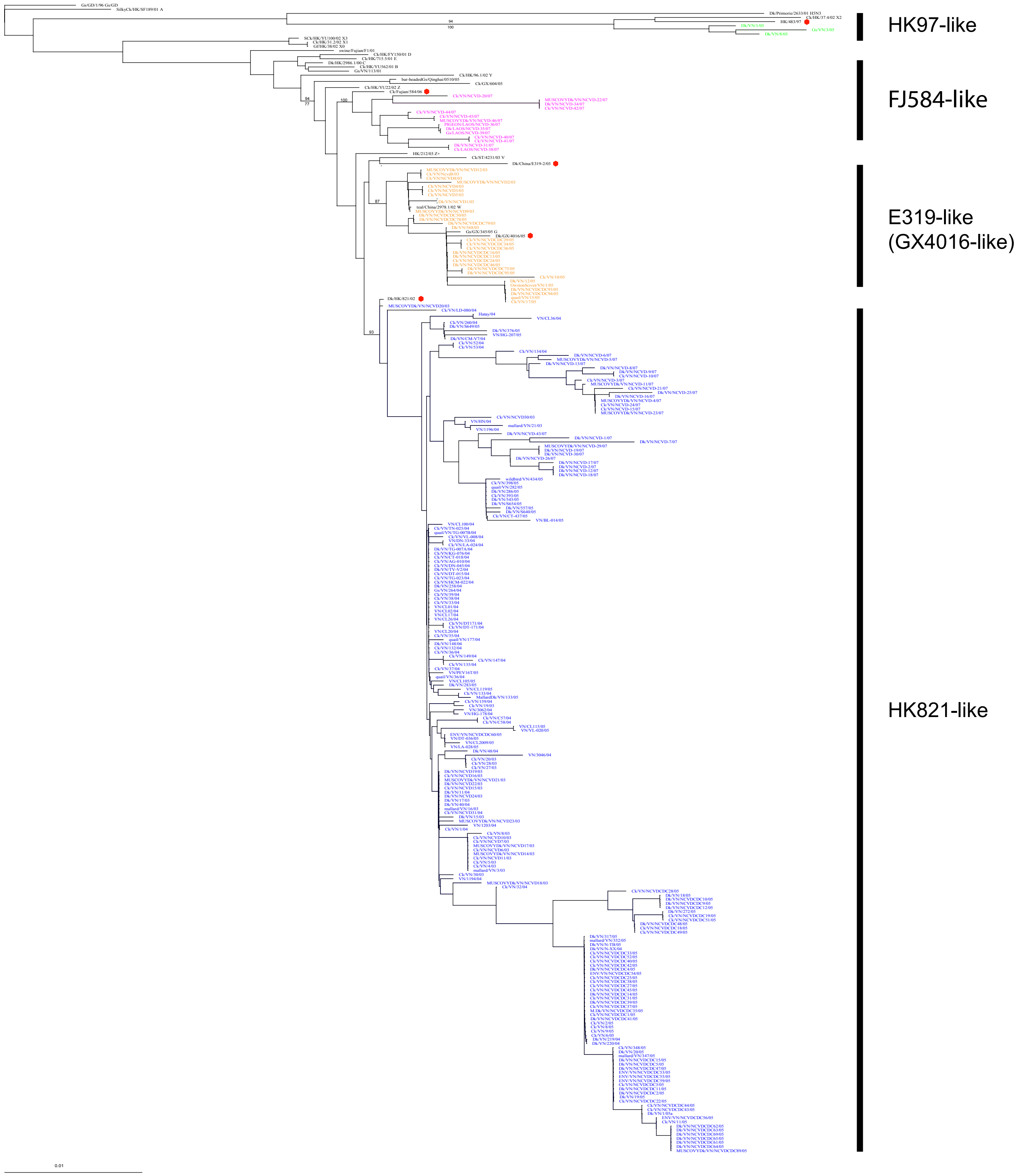

Figure S1H NS

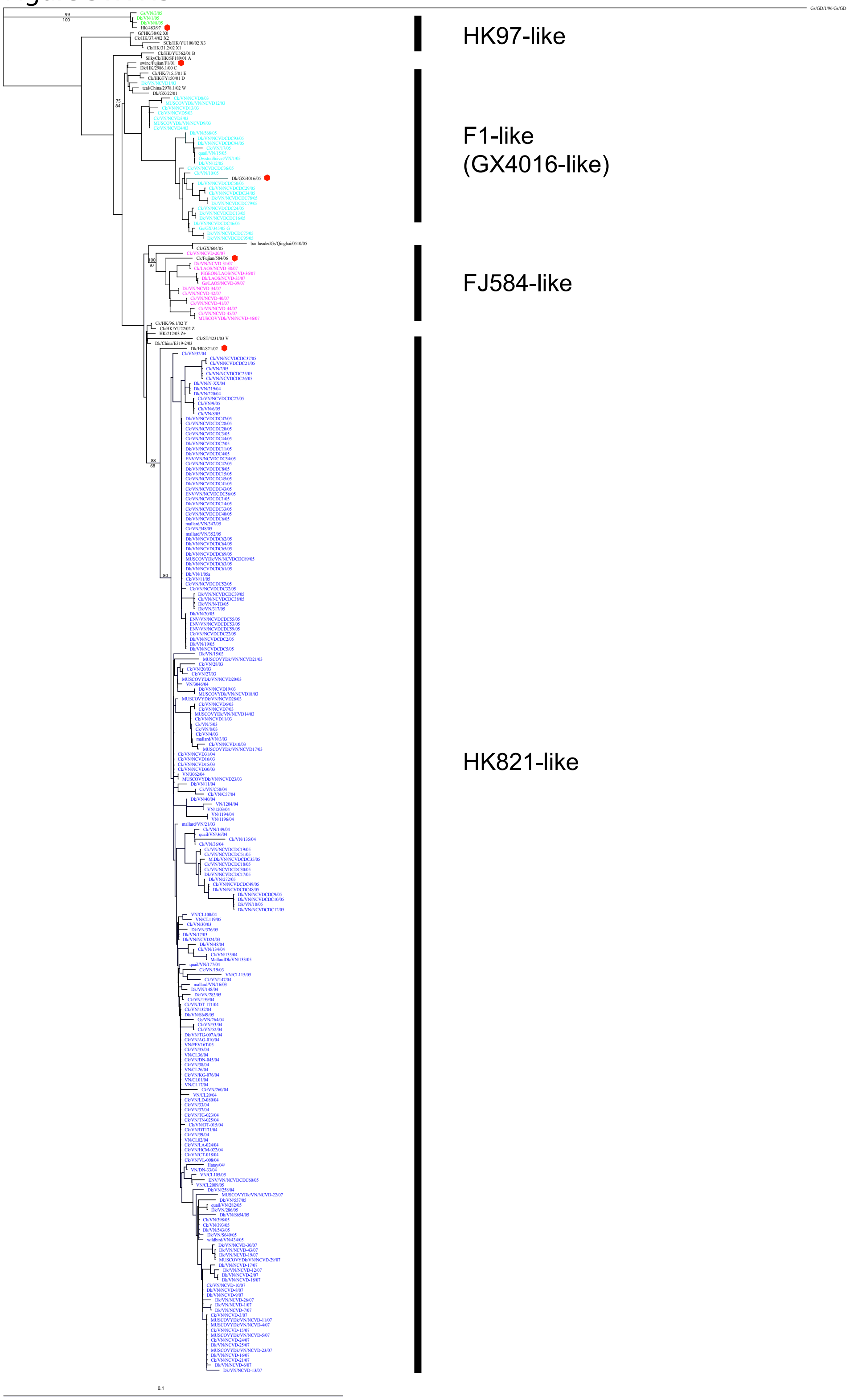

Supplement: Figure S1 — Phylogenetic analysis of the H5N1 highly pathogenic avian influenza viruses isolated in Vietnam. Figures A-H represent segments HA, NA, PB2, PB1, PA, NP, MP, and NS, respectively. Posterior probabilities and bootstrap values are given above and below branches. A red dot was marked beside each predicted precursor virus. Previously reported genotypes, such as A, B, C, D, E, G, X0, X1, X2, X3, Y, V, Z, and Z+ [14], [16], [22], and their representative strains were specifically annotated after the strain names. The phylogenetic trees were constructed by Maximum Likelihood using GARLI version 0.951 by selecting the GTR+I+G model from Modeltest 3.7. Trees were unrooted. (3.38 MB PDF) [file pone.0003462.s004.pdf]
