# Supplementary figures and images for: Evolution of Highly Pathogenic H5N1 Avian Influenza Viruses in Vietnam between 2001 and 2007
Source: PLoS One. 2008 Oct 21;3(10):e3462. doi: 10.1371/journal.pone.0003462 (PMC2565130; doi:10.1371/journal.pone.0003462)

Figure S2

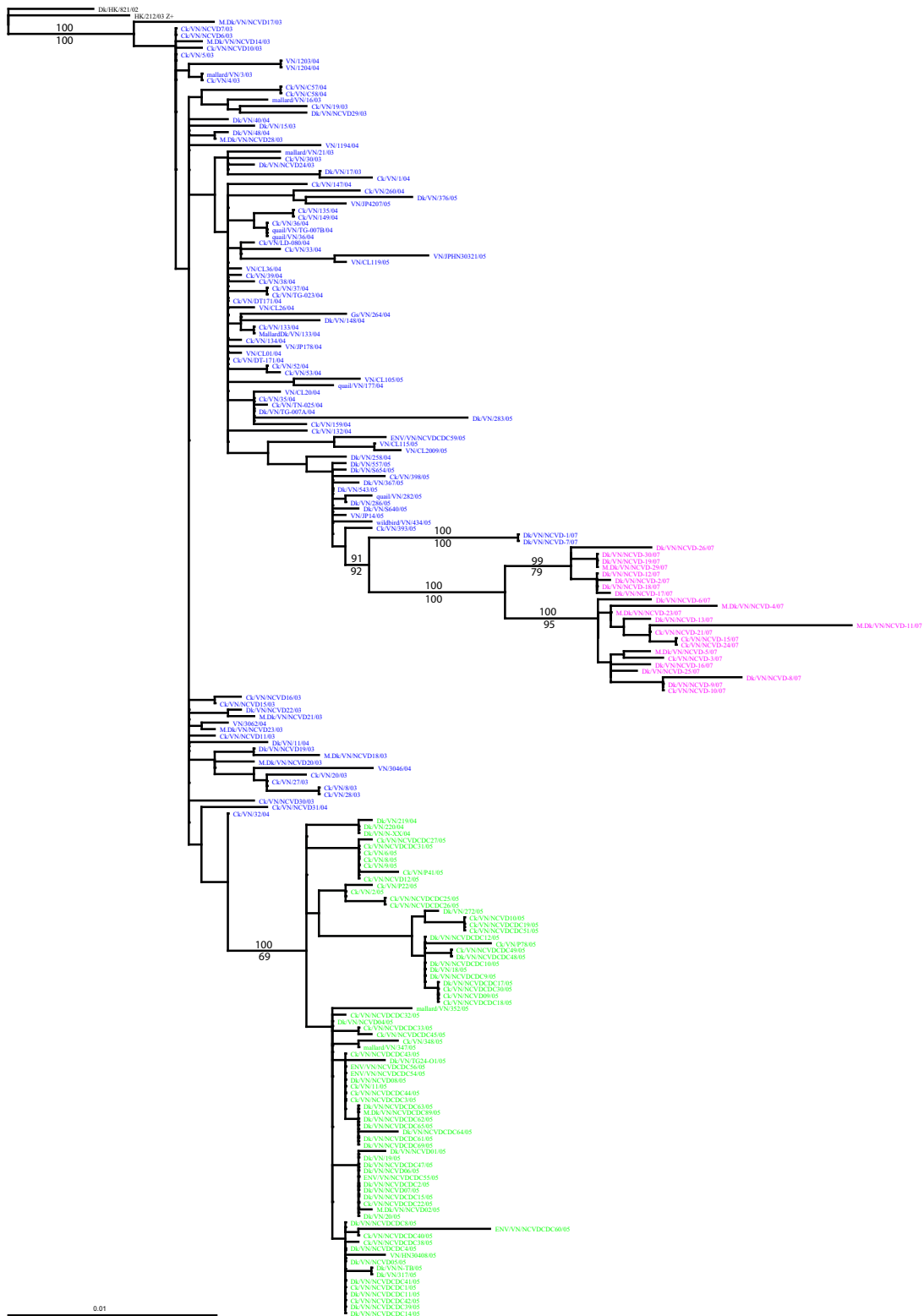

Supplement: Figure S2 — Phylogenetic tree of the HA gene of HK821-like avian influenza viruses isolated from Vietnam. HK821-like viruses formed three sub-lineages: HK821P, HK821α, and HK821β. The tree was rooted by Dk/HK/821/02. The phylogenetic tree was constructed by Maximum Likelihood using GARLI version 0.951 by selecting GTR+I+G model from Modeltest 3.7. Posterior probabilities and bootstrap values were given above and below branches, respectively. (0.11 MB PDF) [file pone.0003462.s005.pdf]
